# Hepatic Deletion of Janus Kinase 2 Counteracts Oxidative Stress in Mice

**DOI:** 10.1038/srep34719

**Published:** 2016-10-07

**Authors:** Madeleine Themanns, Kristina M. Mueller, Sonja M. Kessler, Nicole Golob-Schwarzl, Thomas Mohr, Doris Kaltenecker, Jerome Bourgeais, Jamile Paier-Pourani, Katrin Friedbichler, Doris Schneller, Michaela Schlederer, Eva Zebedin-Brandl, Luigi M. Terracciano, Xiaonan Han, Lukas Kenner, Kay-Uwe Wagner, Wolfgang Mikulits, Andrey V. Kozlov, Markus H. Heim, Fabrice Gouilleux, Johannes Haybaeck, Richard Moriggl

**Affiliations:** 1 Ludwig Boltzmann Institute for Cancer Research, Vienna, Austria; 2 Institute of Animal Breeding and Genetics, University of Veterinary Medicine, Vienna, Austria; 3 Department of Pharmacy, Pharmaceutical Biology, Saarland University, Saarbrücken, Germany; 4 Center for Biomarker Research in Medicine, Graz, Austria; 5 Institute of Cancer Research, Department of Medicine I, Comprehensive Cancer Center, Medical University of Vienna, Vienna, Austria; 6 François Rabelais University, CNRS UMR 7292, LNOx team, Tours, France; 7 Ludwig Boltzmann Institute for Experimental and Clinical Traumatology in AUVA Center, Vienna, Austria; 8 Institute of Clinical Pathology, Medical University of Vienna, Vienna, Austria; 9 Institute of Pharmacology, Centre of Physiology and Pharmacology, Medical University of Vienna, Vienna, Austria; 10 Molecular Pathology Division, Institute of Pathology, University Hospital of Basel, Basel, Switzerland; 11 Division of Gastroenterology, Hepatology and Nutrition, Cincinnati Children’s Hospital Medical Center, Cincinnati, OH, USA; 12 Unit of Pathology of Laboratory Animals, University of Veterinary Medicine, Vienna, Austria; 13 Eppley Institute for Research in Cancer and Allied Diseases, University of Nebraska Medical Center, Omaha, USA; 14 Department of Biomedicine, University Hospital Basel, Basel, Switzerland; 15 Institute of Pathology, Medical University of Graz, Graz, Austria; 16 Medical University of Vienna, Vienna, Austria

## Abstract

Genetic deletion of the tyrosine kinase JAK2 or the downstream transcription factor STAT5 in liver impairs growth hormone (GH) signalling and thereby promotes fatty liver disease. Hepatic STAT5 deficiency accelerates liver tumourigenesis in presence of high GH levels. To determine whether the upstream kinase JAK2 exerts similar functions, we crossed mice harbouring a hepatocyte-specific deletion of JAK2 (JAK2^Δhep^) to GH transgenic mice (GH^tg^) and compared them to GH^tg^STAT5^Δhep^ mice. Similar to GH^tg^STAT5^Δhep^ mice, JAK2 deficiency resulted in severe steatosis in the GH^tg^ background. However, in contrast to STAT5 deficiency, loss of JAK2 significantly delayed liver tumourigenesis. This was attributed to: (i) activation of STAT3 in STAT5-deficient mice, which was prevented by JAK2 deficiency and (ii) increased detoxification capacity of JAK2-deficient livers, which diminished oxidative damage as compared to GH^tg^STAT5^Δhep^ mice, despite equally severe steatosis and reactive oxygen species (ROS) production. The reduced oxidative damage in JAK2-deficient livers was linked to increased expression and activity of glutathione S-transferases (GSTs). Consistent with genetic deletion of *Jak2*, pharmacological inhibition and siRNA-mediated knockdown of *Jak2* led to significant upregulation of *Gst* isoforms and to reduced hepatic oxidative DNA damage. Therefore, blocking JAK2 function increases detoxifying GSTs in hepatocytes and protects against oxidative liver damage.

Non-alcoholic fatty liver disease (NAFLD) is becoming the most common chronic liver disease and represents an enormous public health problem. Accumulation of ectopic lipids in liver is the hallmark of NAFLD. Its histologic spectrum ranges from simple steatosis to the progressive form of non-alcoholic steatohepatitis (NASH), which might further progress to cirrhosis and its related complications, such as hepatocellular carcinoma (HCC)^1^,^2^,^3^. There is evidence that HCC can develop in NAFLD patients even without cirrhosis, suggesting an association between abnormal metabolic processes and HCC^2^,^4^,^5^,^6^. Increased generation of reactive oxygen species (ROS) is found in patients and mouse models with NAFLD^6^,^7^,^8^,^9^,^10^,^11^. When the equilibrium between ROS generation and the antioxidant defence is disrupted, the resulting oxidative stress promotes liver injury, which increases the risk for HCC development^7^.

Growth hormone (GH) affects whole-body physiology via the widely expressed GH receptor (GHR). In the liver GH activates the Janus kinase (JAK) 2 and signal transducer and activator of transcription (STAT) 5 signalling pathway to regulate target genes involved in vital liver functions^12^. GH-deficient or GH-resistant states both cause impaired signal transduction through the GHR in liver and this correlates with chronic liver disease including NAFLD^13^,^14^,^15^,^16^. Similarly, mice lacking components of the GHR-JAK2-STAT5 axis in hepatocytes develop hepatic GH resistance and steatosis^17^,^18^,^19^,^20^,^21^. We and others identified hepatic STAT5 deficiency as being associated with higher susceptibility to liver cancer^20^,^22^,^23^,^24^, driven through progressive steatosis.

Here, we addressed the impact of hepatic JAK2 deficiency on the progression of hepatic steatosis. Furthermore, we aimed to elucidate the contribution of hepatic JAK2 and STAT5 deficiency to oxidative stress with consequence for HCC development in a situation of hyperactived GH signalling. Therefore, we crossed mice harbouring a hepatocyte-specific deletion of either JAK2 or STAT5 (JAK2^Δhep^, STAT5^Δhep^; using AlfpCre^24^,^25^) to growth hormone transgenic mice (GH^tg^)^26^. GH^tg^ mice display high systemic circulating levels of GH that are causally related to a sustained increase in hepatocyte turnover and hyperplasia followed by development of hepatocellular tumours^26^.

Consistent with previous findings^18^,^21^, JAK2 deficiency caused hepatic steatosis by inducing lipid redistribution to the liver and hepatic lipid synthesis. Yet, despite profound steatosis in GH^tg^JAK2^Δhep^ mice, liver tumourigenesis was delayed as compared to GH^tg^STAT5^Δhep^. While accelerated tumourigenesis in STAT5-deficient mice was associated with aberrant activation of STAT3 and increased oxidative damage, delayed carcinogenesis in JAK2-deficient mice was linked to reduced STAT3 signalling and ROS-induced oxidative damage.

## Results

### JAK2 deficiency promotes hepatic steatosis by ectopic lipid redistribution and *de novo* lipogenesis

**Figure 1:**
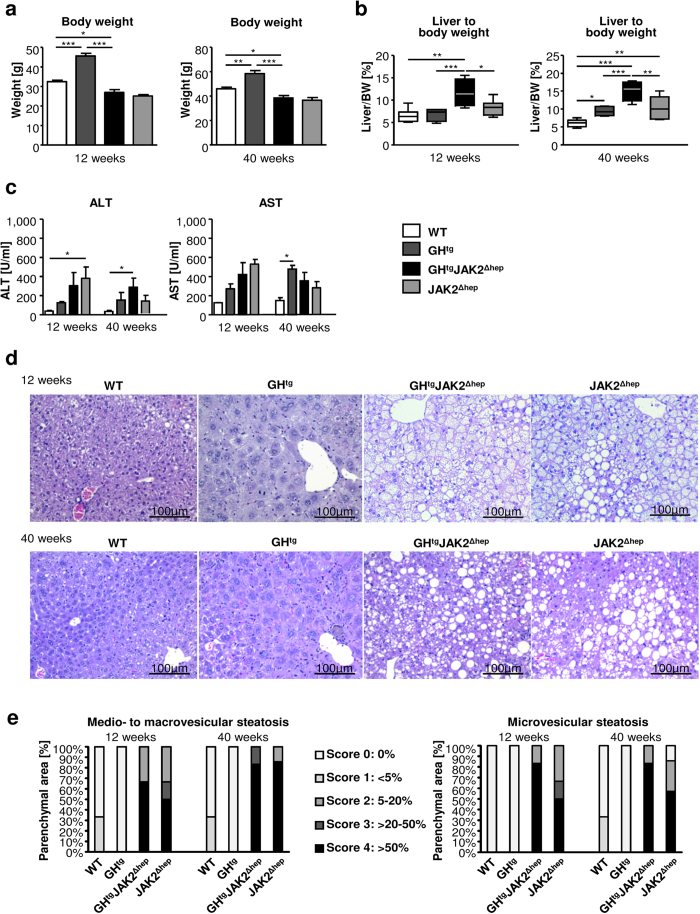
Loss of hepatic JAK2 leads to profound hepatic steatosis. (**a**) Body weights of mice at 12 and 40 weeks of age (n ≥ 4/genotype for each time point) (**b**) Liver weight (LW)/body weight (BW) ratio of mutant and WT mice at indicated time points (n ≥ 4/genotype). (**c**) Liver damage was quantified by measuring serum levels of the liver enzymes ALT and AST at indicated time points (n ≥ 4/genotype). (**d**) H&E staining of liver sections at indicated time points. (**e**) Histopathological analysis assessing medio- and microvesicular steatosis (n ≥ 4/genotype). *p < 0.05, **p < 0.01 and ***p < 0.001.

JAK2 deficiency alone and in combination with hyperactivated GH signalling resulted in severe hepatic steatosis and a reduction in body weight, which is consistent with previous reports^18^,^21^. The reduction in body weight remained at approximately 20% in both JAK2-deficient lines compared to WT mice at 12 and 40 weeks of age, while body weights of GH^tg^ mice were increased (Fig. 1a). Hepatomegaly was already evident in 12-week old GH^tg^JAK2^Δhep^ animals. At 40 weeks of age, GH^tg^JAK2^Δhep^ mice displayed increased liver weight (LW) to body weight (BW) ratios of 15% compared to WT mice, while LW/BW ratios of GH^tg^ and JAK2^Δhep^ mice were elevated by 10% (Fig. 1b). Furthermore, in comparison to WT mice, serum levels of alanine aminotransferase (ALT) and aspartate-aminotransferase (AST) were elevated in all genotypes as indicators of liver damage (Fig. 1c). Subsequent histopathological analysis confirmed severe steatosis in both GH^tg^JAK2^Δhep^ and JAK2^Δhep^ mice characterised by medio- to macro- and microvesicular steatosis (Fig. 1d), which was accompanied by mild lobular inflammation at 40 weeks of age when compared to WT littermates (Supplementary Fig. S1a). Particularly, microvesicular steatosis was more prominent in livers of young and aged GH^tg^JAK2^Δhep^ mice (Fig. 1e, Supplementary Fig. S1a). Electron microscopy analysis revealed big and swollen mitochondria in GH^tg^JAK2^Δhep^ and JAK2^Δhep^ livers (Supplementary Fig. S1b), indicating mitochondrial dysfunction and fatty degeneration in hepatocytes.

**Figure 2:**
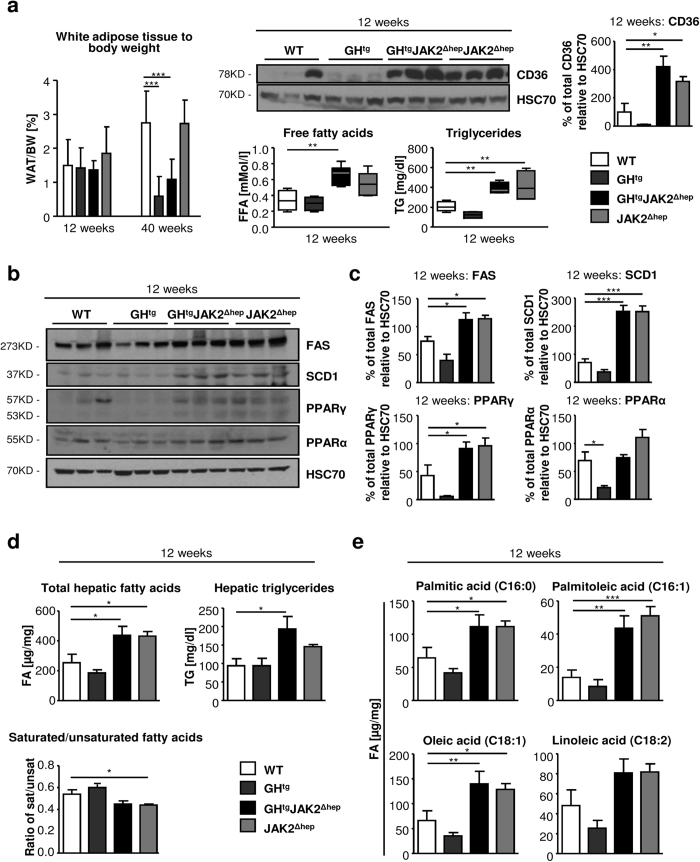
JAK2 deficiency leads to increased mobilisation of fatty acids from the periphery and hepatic *de novo* lipogenesis. (**a**) WAT/body weight ratio of perigonadal fat tissue indicating lipid mobilisation at indicated time points (n ≥ 4/genotype). Representative Western blot analysis of whole liver homogenates and Western blot quantification of CD36 from 12-week-old animals. HSC70 is shown as loading control (n = 3/genotype). Scans of blots are presented in Supplementary Fig. S8. At 12 weeks of age, FFA serum levels, which were determined using an enzymatic test, were elevated in all mice lacking JAK2 (n ≥ 5/genotype). Serum TG levels were measured at 12 weeks of age (n ≥ 4/genotype). (**b**) Representative Western blot analysis of whole liver homogenates from 12-week-old animals. As a loading control HSC70 is shown (n = 3/genotype). (**c**) Western blot quantification of total FAS, SCD1, PPARγ and PPARα. Scans of blots are presented in Supplementary Fig. S8. (**d**) Hepatic total FA and TG amounts were measured at 12 weeks of age (n ≥ 6/genotype). Ratio of saturated/unsaturated hepatic FAs at 12 weeks of age (n ≥ 8/genotype). (**e**) Detailed profiling of steatotic livers using gas chromatography–mass spectrometry (GC-MS) showed elevated unsaturated FAs at 12 weeks of age (n ≥ 8/genotype). *p < 0.05, **p < 0.01 and ***p < 0.001.

Next, we investigated the consequences of JAK2 deficiency for lipid metabolism. In GH^tg^JAK2^Δhep^ animals, high GH levels led to massive lysis of perigonadal white adipose tissue (WAT), which was not affected in JAK2^Δhep^ mice (Fig. 2a). Lipolysis was manifested by a reduction of WAT/BW ratios over time and an increase in circulating free fatty acids (FFAs). This coincided with greatly elevated protein levels of FA translocase CD36 in JAK2^Δhep^ and GH^tg^JAK2^Δhep^ livers, indicating an increased capability of hepatic FFA uptake^18^,^27^. Furthermore, significantly elevated serum triglyceride (TG) levels, were found in JAK2^Δhep^ and GH^tg^JAK2^Δhep^ mice (Fig. 2a), indicative of hypertriglyceridemia^28^. Hepatic steatosis observed in both JAK2-deficient lines was not only a result of ectopic FA uptake but also resulted from enhanced expression of key enzymes involved in *de novo* lipogenesis. Lipogenic proteins like fatty acid synthase (FAS), stearoyl-CoA desaturase (SCD) 1, and peroxisome proliferator-activated receptor gamma (PPARγ) were significantly upregulated as verified with Western blot quantification in both JAK2-deficient lines (Fig. 2b,c). Protein levels of PPARα, a major regulator of catabolism of FAs, were not changed in JAK2-deficient livers (Fig. 2b,c). In addition, detailed profiling of steatotic livers at 12 and 40 weeks of age revealed increased total FA and TG amounts characterised by significantly elevated unsaturated FAs like palmitoleic acid and oleic acid (Fig. 2d,e; Supplementary Fig. S2a–c). Similar results have been described for GH^tg^STAT5^Δhep^ mice where profound steatosis was a result of increased WAT mobilisation and the induction of hepatic *de novo* lipogenesis^24^. Collectively, these data indicate that a combination of increased mobilisation of WAT and the induction of hepatic *de novo* lipogenesis contributes to the steatotic phenotype of both JAK2-deficient lines.

### Liver tumourigenesis is delayed upon loss of JAK2 in GH^tg^ mice

**Figure 3:**
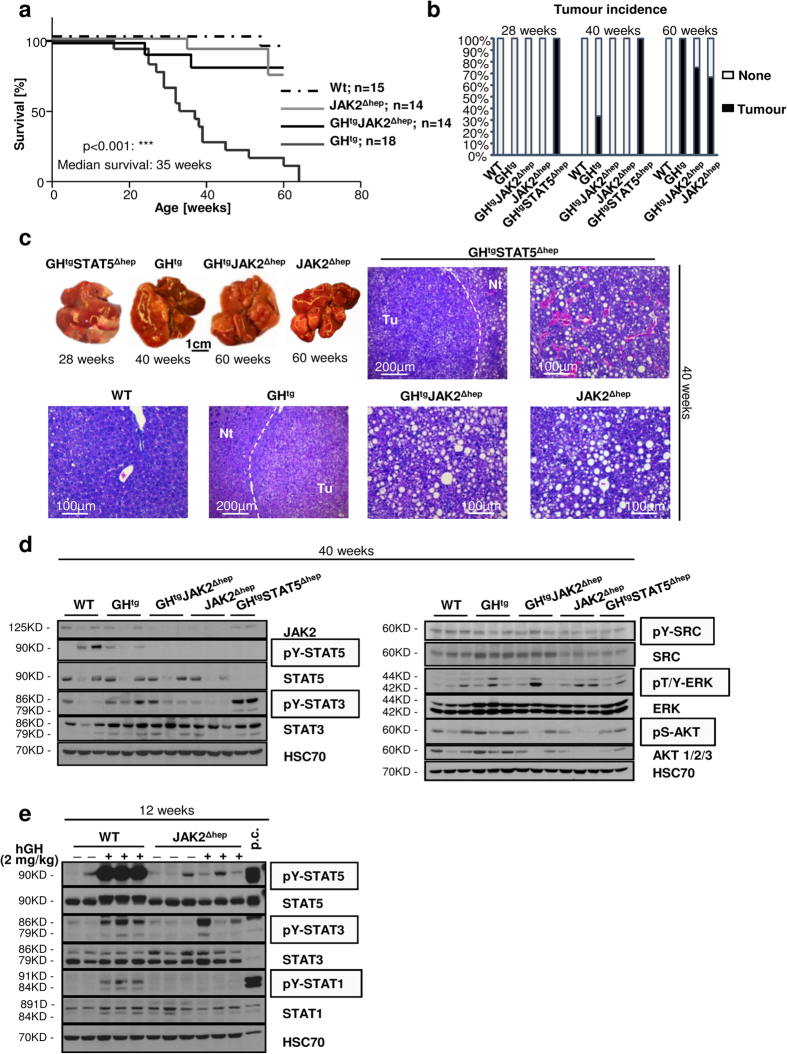
Liver tumourigenesis is enhanced in STAT5-deficient animals, but diminished upon loss of its upstream kinase JAK2. (**a**) Kaplan-Meier plot of male mice of four distinct genotypes over a time period of 60 weeks (n ≥ 14/genotype). (**b**) Tumour incidence of livers of GH^tg^STAT5^Δhep^, GH^tg^, GH^tg^JAK2^Δhep^ and JAK2^Δhep^ mice. At 40 weeks of age, GH^tg^ mice developed first liver tumours. Additional deletion of STAT5 in hepatocytes led to earlier and malignant tumours, whereas deletion of JAK2 resulted in delayed tumour formation at 60 weeks of age (n ≥ 4/genotype). (**c**) Macroscopic appearance of tumourigenic livers at indicated time points. At 40 weeks, GH^tg^STAT5^Δhep^ mice had large steatotic and solid tumours. Tumours displayed a solid or trabecular growth pattern (n ≥ 4/genotype). (**d**) Representative Western blot analysis of oncogenic signalling pathways in whole liver homogenates at 40 weeks of age. HSC70 is shown as loading control (n ≥ 2/genotype). Scans of blots are presented in Supplementary Fig. S9. (**e**) Representative Western blot analysis of STAT proteins. As a loading control HSC70 is shown. WT and JAK2^Δhep^ animals were injected intraperitoneally with vehicle or 2 mg/kg hGH and sacrificed 30 minutes thereafter (n = 3/treatment). Scans of blots and different exposure times of pY-STAT5 are presented in Supplementary Fig. S9. ***p < 0.001.

High systemic levels of GH significantly reduce the life expectancy of GH^tg^ mice^24^,^29^ which is corrected upon hepatic STAT5^24^ or JAK2 deficiency (Fig. 3a). We and others reported that hepatic STAT5 deficiency promotes liver tumour development and progression^22^,^23^,^24^. At 28 weeks of age, all GH^tg^STAT5^Δhep^ animals succumbed to liver tumours, which were characterised by broadening of liver cell cords and the loss of lobular plate architecture, pronounced cellular pleomorphisms and solid or trabecular growth pattern^24^. In contrast, GH^tg^ mice developed first tumours 12 weeks later with an incidence rate of 34% (Fig. 3b,c). At this time point 100% of GH^tg^JAK2^Δhep^ and JAK2^Δhep^ livers remained tumour-free (Fig. 3b,c), despite similar proliferation rates of hepatocytes (Supplementary Fig. S3a). The occurrence of liver tumours in GH^tg^JAK2^Δhep^ and JAK2^Δhep^ mice was observed 5 months later with an incidence of 76% and 68%, respectively. Histopathological analysis revealed that GH^tg^JAK2^Δhep^ and JAK2^Δhep^ tumours both displayed cellular polymorphism and expression of markers for hepatocellular malignancy, such as oval cell fraction, and eosinophilic inclusions (Supplementary Fig. S3b).

Aberrant GH-mediated activation of STAT3 contributes to liver tumourigenesis in STAT5-deficient mice^19^,^20^,^24^, which was clearly prevented in JAK2-deficient livers (Fig. 3d). In agreement, GH treatment of JAK2^Δhep^ mice did not result in activation of STAT1/3/5 (Fig. 3e). Notably, not all GHR responsive signalling pathways require JAK2 activity^30^; these include GH dependent activation of SRC and ERK1/2, both of which can exert oncogenic functions in liver. However, neither SRC nor ERK1/2 was aberrantly activated in livers of GH^tg^JAK2^Δhep^ and GH^tg^STAT5^Δhep^ mice (Fig. 3d), suggesting that these signalling pathways do not contribute to liver tumourigenesis in either model. These results demonstrate that hepatic JAK2 deficiency - in contrast to STAT5 deletion - delays tumour formation in the GH transgenic background.

### Loss of JAK2 protects against ROS-induced oxidative damage

**Figure 4:**
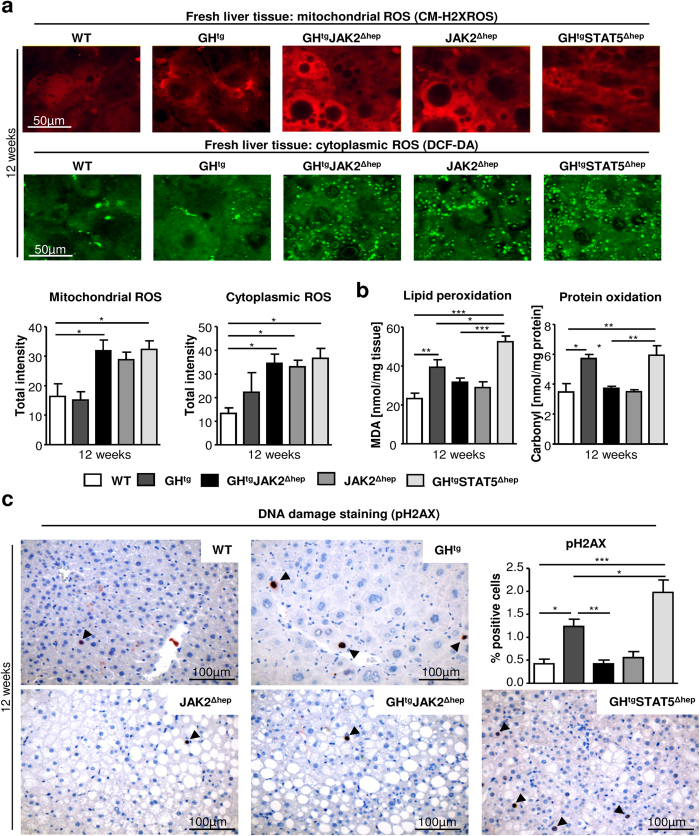
Hepatic JAK2 deficiency protects against ROS-induced lipid peroxidation, protein oxidation and DNA damage. (**a**) Freshly cut livers were stained with fluorescent dyes sensitive to mitochondrial and cytoplasmic ROS. Imaging was performed with an inverted confocal microscope (n ≥ 5/genotype). (**b**) Lipid peroxidation was assessed by measuring its by-product malondialdehyde (MDA) at 12 weeks of age. Measurement of protein oxidation was performed using colorimetric assay (n ≥ 5/genotype). (**c**) Representative liver sections of 12-week-old mice stained with antibodies against phosphorylated H2AX to detect DNA damage. Quantification of positive hepatocytes by image analysis (n ≥ 5/genotype). *p < 0.05, **p < 0.01 and ***p < 0.001.

Oxidative stress is a common feature of NAFLD and the resulting cellular damage (i.e. lipid peroxidation, protein oxidation and DNA damage)^8^ contributes to liver tumourigenesis. To investigate the contribution of oxidative stress to progression of steatosis upon hepatic JAK2 or STAT5 deficiency, we determined ROS production within hepatocytes in 12-week-old animals by using different dyes, which are sensitive to mitochondrial membrane potential, mitochondrial or cytoplasmic ROS. For this purpose liver tissues were freshly excised, sectioned and subsequently stained. Mitochondrial membrane potential was similar among the genotypes, while mitochondrial and cytoplasmic ROS was increased in hepatocytes of steatotic GH^tg^STAT5^Δhep^, GH^tg^JAK2^Δhep^ and JAK2^Δhep^ livers (Fig. 4a, Supplementary Fig. S4a). Cytoplasmic ROS in GH^tg^JAK2^Δhep^, JAK2^Δhep^ and GH^tg^STAT5^Δhep^ livers was recognised as small subcellular structures indicative for peroxisome activation. Surprisingly, despite similarly elevated ROS levels, increased DNA damage (pH2AX), protein carbonylation (indicative for protein oxidation), and malondialdehyde (MDA), a side product of lipid peroxidation, were only significantly increased in GH^tg^STAT5^Δhep^ and GH^tg^ animals at 12 and 40 weeks of age (Fig. 4b,c; Supplementary Fig. S4b,c). These data indicate that hepatic JAK2 deficiency protects against ROS-induced DNA damage and oxidation of lipids and proteins, while STAT5 deficiency results in accelerated oxidative damage.

### Loss of JAK2 leads to increased detoxification by glutathione S-transferases

**Figure 5:**
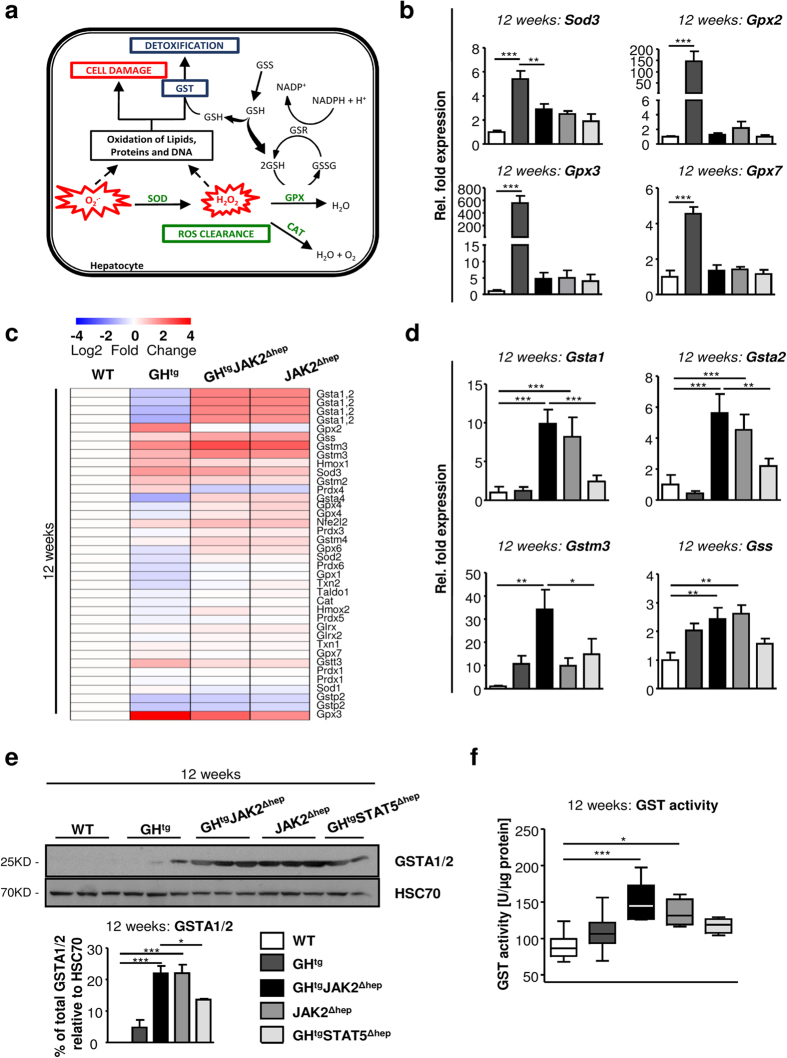
Genetic deletion of hepatic *Jak2* results in increased expression and activity of detoxifying enzymes. (**a**) Schematic overview of the antioxidative defence system. SOD, CAT and GPX are the primary antioxidant enzymes which inactivate ROS into intermediates. GST catalyses the conjugation of the reduced form of GSH to oxidised products to detoxify oxidised lipids, proteins and DNA (H^+^: hydrogen ion, SOD: superoxide dismutase, CAT: catalase, GPX: glutathione peroxidase, GSSG: oxidized glutathione, GSH: glutathione, GSS: glutathione synthetase, GSR: glutathione reductase, GST: glutathione S-transferase). (**b**) By means of qRT-PCR, mRNA levels of *Sod3*, *Gpx2*, *Gpx3* and *Gpx7* involved in antioxidant response were measured in livers at 12 weeks of age (n = 6/genotype). Ct values were normalised to *Gapdh* and *Rpl12a*. (**c**) At 12 weeks of age transcriptome analysis of genes coding for enzymes involved in antioxidant defence. (**d**) mRNA expression levels of *Gsta1*, *Gsta2, Gstm3* and *Gss* at 12 weeks of age (n = 6/genotype). Ct values were normalised to *Gapdh*. (**e**) Representative Western blot analysis of whole liver homogenates and Western blot quantification of GSTA1/2 from 12-week-old animals. As a loading control HSC70 is shown (n ≥ 2/genotype). Scans of blots are presented in Supplementary Fig. S10. (**f**) GST activity was assessed in livers from 12-week-old mice using a colorimetric assay (n ≥ 6/genotype). *p < 0.05, **p < 0.01 and ***p < 0.001.

Protection from ROS-mediated oxidative damage is conducted by several defence mechanisms, such as ROS clearance and the detoxification machinery^9^. Inadequate defence mechanisms result in cell damage and thereby, increase the risk for malignant transformation (Fig. 5a). Thus, to define the mechanisms underlying the different degrees of oxidative damage upon hepatic JAK2 or STAT5 deficiency, we examined the expression levels of enzymes involved in ROS clearance and detoxification. Global gene-expression analysis of enzymes governing ROS clearance revealed that the expression of superoxide dismutase 3 (*Sod3*), glutathione peroxidase 2 (*Gpx2*), *Gpx3* and *Gpx7* was highly upregulated in GH^tg^ animals at 12 weeks of age (Fig. 5b,c; Supplementary Fig. S5a). SOD3, GPX2 and GPX3 execute extracellular functions and have been shown to exert anti-inflammatory effects^31^,^32^. However, expression levels were not altered in steatotic GH^tg^STAT5^Δhep^, GH^tg^JAK2^Δhep^ and JAK2^Δhep^ livers compared to WT littermates. The second major antioxidant defence mechanism is executed by glutathione-S transferases (GST), which directly detoxify oxidized macromolecules to prevent cell damage (Fig. 5a). At 12 and 40 weeks of age, Affymetrix and qRT-PCR analyses of whole liver extracts revealed strong upregulation of *Gsta1*, *Gsta2* and *Gstm3* particularly in JAK2-deficient livers (Fig. 5c,d; Supplementary Fig. S5b,c). Additionally, the expression of glutathione synthetase (*GSS*), which is necessary for glutathione biosynthesis and GST functionality, was increased in GH^tg^JAK2^Δhep^ and JAK2^Δhep^ mice (Fig. 5d). In line, protein levels of GSTA1/2 and GST activity were significantly elevated in GH^tg^JAK2^Δhep^ and JAK2^Δhep^ livers (Fig. 5e,f; Supplementary Fig. S5d). Taken together, these results suggest that loss of hepatic JAK2 diminishes ROS-induced oxidative damage through increased expression and activity of GST enzymes.

### Pharmacological inhibition and siRNA-mediated knockdown of *Jak2* leads to increased expression of *Gst* isoforms and reduced DNA damage

**Figure 6:**
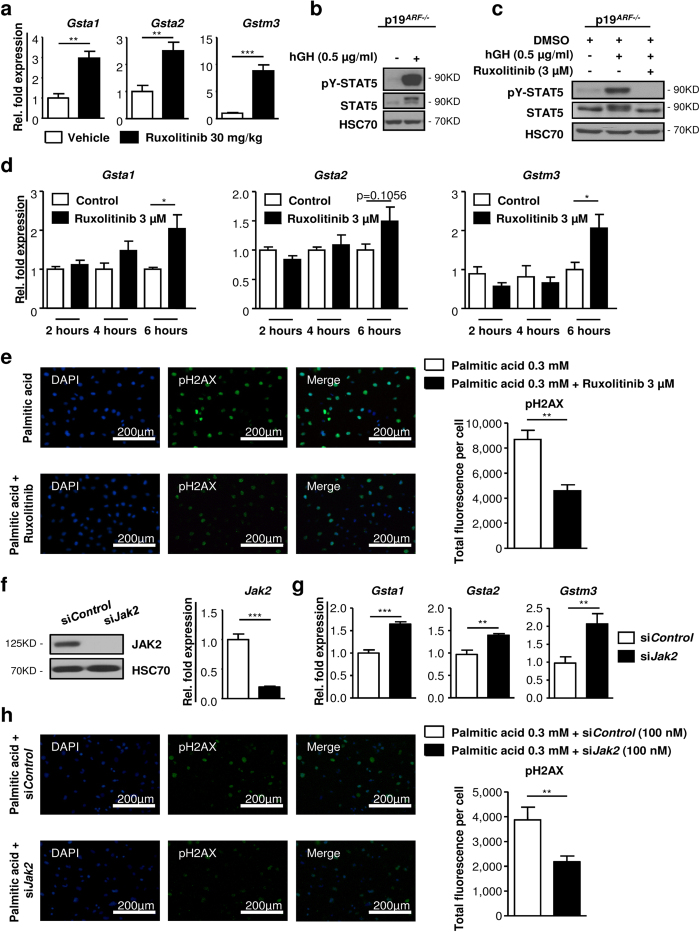
Pharmacological inhibition and siRNA-mediated knockdown of *Jak2* induces expression of *Gst* isoforms and reduces DNA damage upon oxidative stress. (**a**) Control littermates not expressing the AlfpCre were treated with vehicle or 30 mg/kg ruxolitinib twice daily for 2 days by oral gavage (n = 4/treatment). mRNA expression levels of *Gsta1*, *Gsta2* and *Gstm3* upon ruxolitinib treatment. Ct values were normalised to *Gapdh*. (**b**) Representative Western blot analysis showed activation of STAT5 in immortalised p19^ARF−/−^ hepatocytes upon human GH stimulation (0.5 μg/ml). Prior to stimulation, hepatocytes were starved for 2 hours (1% FCS and without growth factors). As a loading control HSC70 is shown (n = 3 independent experiments). Scans of blots are presented in Supplementary Fig. S11. (**c**) After treatment with 3 μM ruxolitinib for 24 hours, p19^ARF−/−^ hepatocytes were stimulated with 0.5 μg/ml of human GH and analysed by Western blotting. As a loading control HSC70 is shown (n = 3 independent experiments). Scans of blots are presented in Supplementary Fig. S11. (**d**) p19^ARF−/−^ hepatocytes were treated with 3 μM ruxolitinib without hGH for indicated time points. mRNA expression levels of *Gsta1*, *Gsta2* and *Gstm3* were measured. Results are shown from three independent experiments. Ct values were normalised to *Gapdh*. (**e**) Immunofluorescence staining of DAPI (blue) and phosphorylated H2AX (green). p19^ARF−/−^ hepatocytes were pre-treated 24 hours with ruxolitinib or DMSO. Cells were exposed with 0.3 mM palmitic acid (PA) for another 24 hours containing either ruxolitinib or DMSO. Fluorescence intensity was quantified by image analysis (n = 3 independent experiments). (**f**) p19^ARF−/−^ hepatocytes were transfected with non-targeting siRNA (si*Control*) or siRNA against *Jak2* (si*Jak2*) for 48 hours. Representative Western blot analysis and mRNA expression levels showed efficient knockdown in p19^ARF−/−^ hepatocytes. As a loading control HSC70 is shown. Scans of blots are presented in Supplementary Fig. S11. (**g**) p19^ARF−/−^ hepatocytes were transfected with si*Jak2* or si*Control* for 48 hours. mRNA expression levels of *Gsta1*, *Gsta2* and *Gstm3* upon siRNA-mediated knockdown were measured. Ct values were normalised to *Gapdh*. (**h**) Immunofluorescence staining of DAPI (blue) and phosphorylated H2AX (green). p19^ARF−/−^ hepatocytes were pre-treated 48 hours with si*Jak2* or si*Control*. Cells were exposed with 0.3 mM PA for another 24 hours. Fluorescence intensity was quantified by image analysis (n = 2 independent experiments). *p < 0.05, **p < 0.01 and ***p < 0.001.

To verify that loss of JAK2 activity directly accounted for the induction of *Gst* expression, we treated WT littermates with the JAK inhibitor ruxolitinib for 2 days. Ruxolitinib treatment induced strong expression of *Gsta1*, *Gsta2* and *Gstm3*, resembling the hepatic knockout of *Jak2*. Liver sections exhibited normal liver architecture in ruxolitinib-treated mice (Fig. 6a; Supplementary Fig. S6a). To assess whether *Gst* expression was hepatocyte-specific, we made use of immortalised murine p19^ARF−/−^ hepatocytes^33^, which were characterised by formation of monolayers, retention of morphological features and the expression of hepatocyte-specific markers as verified by Western blotting (Supplementary Fig. S6b). p19^ARF−/−^ hepatocytes were responsive to GH as indicated by STAT5 activation (Fig. 6b) and JAK2-STAT5 signalling could be completely inhibited by ruxolitinib treatment (Fig. 6c). Importantly, already after 6 hours of JAK2 inhibition, p19^ARF−/−^ hepatocytes displayed increased *Gsta1*, *Gsta2* and *Gstm3* expression (Fig. 6d).

Next, we set out to functionally verify, if inhibition of JAK2 by ruxolitinib would reduce oxidative damage. We generated ROS-induced DNA damage in p19^ARF−/−^ hepatocytes by H_2_O_2_ exposure. Indeed, pre-treatment with ruxolitinib led to a significant reduction of pH2AX expression in hepatocytes (Supplementary Fig. S6c). Similarly, pre-treatment of p19^ARF−/−^ hepatocytes with ruxolitinib resulted in a pronounced reduction of pH2AX expression in hepatocytes upon palmitic acid (PA)-induced lipotoxicity (Fig. 6e)^34^. To exclude potential JAK2 independent effects of ruxolitinib treatment, we additionally made use of small interfering (si)RNA-mediated knockdown of *Jak2*. Complete knockdown in p19^ARF−/−^ hepatocytes was accomplished after 48 hours (Fig. 6f). In accordance with ruxolitinib-treated p19^ARF−/−^ hepatocytes, knockdown of *Jak2* resulted in increased *Gsta1*, *Gsta2* and *Gstm3* expression (Fig. 6g) and reduced ROS-induced DNA-damage after H_2_O_2_ and PA exposure (Fig. 6h; Supplementary Fig. S6d).

Thus, in agreement with genetic loss of JAK2, these data imply that pharmacological inhibition and siNRA-mediated knockdown of *Jak2* reduces oxidative stress-induced DNA damage, presumably, through upregulation of *Gst* enzymes.

Collectively, our data suggest that STAT5 deficiency aggravates liver tumourigenesis in the GH^tg^ background due to aberrant STAT3 activation and increased oxidative damage. In contrast, JAK2 deficiency delays tumour formation in GH^tg^ mice, which is linked to insignificant oncogenic STAT3 signalling and reduced ROS-induced oxidative damage. Our conclusion model (Supplementary Fig. S7) depicts the surprising difference between hepatic STAT5 and JAK2 deficiency with consequence for oxidative liver damage.

## Discussion

Development of NAFLD has been associated with impaired GH signalling^14^,^15^. Mouse models have further linked loss of GH signalling to NAFLD by demonstrating that liver-specific deficiency of GHR, JAK2 or STAT5 results in metabolic defects, which manifest in hepatic steatosis^17^,^18^,^19^. HCC is a common consequence of chronic liver disease^4^,^5^. While JAK2-STAT5 signalling exerts oncogenic functions in hematopoietic cancers^35^, GH-activated STAT5 has a protective role in mouse models of chronic liver disease^12^,^20^,^22^,^23^,^24^,^36^.

Here, we describe that hepatic JAK2 deficiency protects against oxidative liver damage and thereby, potentially reduces the risk for liver tumourigenesis in a GH^tg^ mouse model.

We confirm that loss of JAK2 leads to overt hepatomegaly and steatosis. This phenotype was described to depend on GH-dependent mobilisation of FFAs from WAT and their increased hepatic uptake mediated by elevated expression of PPARγ and CD36^18^,^27^. In contrast to GH^tg^JAK2^Δhep^ mice, JAK2^Δhep^ animals did not display strong increases in circulating FFAs and a depletion of WAT stores. However, even in presence of normal circulating FFA concentrations, transgenic CD36 overexpression in murine liver results in markedly elevated FA uptake and lipid storage^37^. Thus, the greatly increased CD36 expression in JAK2^Δhep^ livers likely promotes hepatic FA uptake and deposition in a similar manner. CD36 is a transcriptional target of PPARγ^38^ and previous work links GH signalling to transcriptional repression of PPARγ^18^. Accordingly, hyperactivated GH signalling, as seen in GH^tg^ mice, suppresses the transcription of PPARγ and its downstream targets, CD36 and SCD1^39^. Conversely, disruption of GH signalling by hepatic deletion of JAK2 or STAT5 results in increased expression of *Cd36*^18^,^19^, which we additionally confirmed by knockdown of *Jak2* in p19^ARF−/−^ hepatocytes (Supplementary Fig. S6e). We extend these findings by demonstrating that hepatic JAK2 deficiency results in elevated protein expression of the lipogenic enzymes FAS and SCD1, both of which are frequently linked to NAFLD^40^,^41^. In agreement with the upregulation of FAS and SCD1^40^,^41^, a specific increase in palmitate (C16:0), palmitoleate (C16:1) and oleate (C18:1) concentrations was observed, suggesting elevated *de novo* synthesis and conversion of saturated FAs into monounsaturated FAs.

A characteristic feature of NAFLD is an increased formation of ROS. Increases in hepatic FA species significantly contribute to ROS production by oxidative events^7^ and thereby to lipotoxicity^42^. Persistent oxidative stress adds to chronic liver injury and this amplifies the risk to develop liver cancer^7^. In line, disruption of GHR-JAK2-STAT5 signalling results in increased production of cytoplasmic and mitochondrial ROS in steatotic hepatocytes^20^. However, despite similar degrees of fatty degeneration and ROS production in GH^tg^STAT5^Δhep^ and GH^tg^JAK2^Δhep^ livers, hepatic JAK2 deficiency delayed tumour formation in the GH^tg^ background. In accordance with our data, increased activation of JAK2 correlates with poor overall survival in HCC patients with high circulating prolactin levels^43^. Furthermore, mice lacking the GHR in a model of cholestasis were largely resistant to tumour development despite an aggravation of liver fibrosis^44^, while a clinical study revealed very low incidence of cancer in a population suffering from a *GHR* mutation^45^. Besides the clinically approved ruxolitinib, there are more than 10 distinct JAK2 inhibitors in clinical trials^46^ emphasising the pharmacological relevance of this pathway.

Aberrant GH-mediated activation of STAT3 was associated with accelerated liver tumourigenesis of STAT5-deficient livers^19^,^20^,^24^. In agreement with the requirement of JAK2 for GH-mediated activation of STAT proteins^30^, excessive STAT3 activity was not observed in JAK2-deficient livers and thus presumably contributed to delayed tumour onset in GH^tg^JAK2^Δhep^ mice. Intriguingly, in the absence of JAK2, elevated ROS accumulation did not correlate with augmented lipid peroxidation, protein oxidation and DNA damage. Several potential therapeutic strategies to lower oxidative stress in NAFLD have been suggested^10^. In particular, vitamin E supplementation is used to decrease TG accumulation and to lower lipid peroxidation^10^,^47^. Along this line, we show that JAK2 deficiency led to increased expression and activity of GSTs, a group of detoxification enzymes involved in protection against oxidative stress^48^. A similar increase in GST expression and activity was not detectable in STAT5-deficient livers, in which elevated ROS accumulation correlated with augmented oxidative damage. A recent study linked STAT5 activation to increased ROS production in chronic myeloid leukaemia cells by repressing antioxidant enzymes suggesting tissue-specific functions of STAT5^49^.

In support of our *in vivo* findings, treatment of p19^ARF−/−^ hepatocytes with ruxolitinib and siRNA against *Jak2* not only resulted in *Gst* upregulation, but was also efficient in reducing H_2_O_2_ and palmitic acid induced oxidative DNA damage. Given the detoxifying role of GST isoforms upregulation in the absence of JAK2 potentially contributes to decreased oxidative damage and delayed tumour initiation in GH^tg^JAK2^Δhep^ mice. The role of GSTs in NAFLD and its progression are not understood. However, a *GSTM1-null* genotype has been implicated in NAFLD development and this finding might be correlated with increased HCC risk in the Asian population^50^. GST activity was further reported to decrease with NAFLD progression accompanied by a reduced pool of glutathione, which inversely correlated with lipid peroxidation^11^. In conjunction, our findings provide evidence that GST enzymes - through resolution of oxidative stress - might be beneficial for the prevention of NAFLD-associated liver damage.

The expression of *Gsts* has been shown to be induced by nuclear factor-like 2 (NRF2), and the nuclear hormone receptors retinoic acid receptor alpha (RARα) and retinoid X receptor alpha (RXRα)^51^. Neither *Nrf2* mRNA expression nor protein levels of RARα and RXRα were significantly upregulated in JAK2-deficient livers (Supplementary Fig. S5e). Interestingly, JAK2 can exert nuclear or peri-nuclear function in hepatocytes, mammary epithelial or hematologic cells^52^,^53^,^54^. This might be a possible mechanism to repress *Gst* induction in the presence of JAK2. However, JAK2-dependent regulation of chromatin architecture identified to date is rather associated with activating than repressive function^52^.

Collectively, we show that ROS-mediated oxidative damage is prevented in steatotic JAK2 deficient livers, which correlates with delayed tumour onset in the GH^tg^ background. Importantly, in line with findings from the genetic model, our data indicate that pharmacologic JAK2 inhibition protects against oxidative damage. Further studies assessing the effectiveness of JAK2 inhibitors to lower oxidative stress may provide a rationale for the potential use in chronic liver disease.

## Methods

### Mice

Mice with hepatic deletion of *Jak2* or *Stat5* (*Jak2*^fl/fl ^25, *Stat5ab*^fl/fl^; referred to as JAK2^Δhep^ or STAT5^Δhep ^55) were bred with GH transgenic animals (GH^tg^; described in ref. 26) to generate GH^tg^JAK2^Δhep^ and GH^tg^STAT5^Δhep^ mice, respectively. Littermates not expressing AlfpCre recombinase served as WT controls. The genetic background of all mice analysed was C57BL/6J × SV129. For all analyses only male mice were used. Animal experiments were performed according to an ethical animal license protocol approved by the authorities of the Austrian government and the Medical University of Vienna. Maintenance and experimental procedures of mice are described in detail in the Supplementary materials and methods (see Supplementary information).

### Serum biochemistry

Serum levels of alanine aminotransferase (ALT) and aspartate-aminotransferase (AST) were determined using the test strip-based Reflotron Plus analyser (Roche). Free fatty acid levels were assessed photometrically using the NEFA-HR(2) kit (Wako).

### Measurement of hepatic metabolites

Hepatic triglycerides (CaymanChemical), lipid peroxidation, protein oxidation and GST activity (BioVision) were determined using commercially available colorimetric assays. The measurement of ROS levels and hepatic fatty acid is described in Supplementary materials and methods (see Supplementary information).

### Immunoblotting and immunohistochemistry

Western blot analyses and immunohistochemistry were performed according to standard protocols. Antibodies used are listed in detail in the Supplementary materials and methods (see Supplementary information).

### Microarray analysis

At 12 and 40 weeks, total RNA from 3 livers/genotype (WT, GH^tg^, GH^tg^JAK2^Δhep^ and JAK2^Δhep^) were isolated using the RNAeasy Kit (#74104; Quiagen) and hybridised to GeneChip Mouse Gene 1.0 ST array (Affymetrix). Microarray data and description of the experimental design is deposited under ArrayExpress, with the accession number: E-MTAB-3774. The detailed analysis is provided in the Supplementary materials and methods (see Supplementary information).

### Analysis of gene expression by quantitative PCR

Total RNA was isolated with TRIzol reagent (Invitrogen) and reverse transcribed using Revert Aid cDNA synthesis kit (Thermo Fisher Scientific). The detailed method together with the primer sequences is provided in the Supplementary materials and methods (see Supplementary information).

### Cell Culture

Hepatocytes of p19^ARF−/−^ mice were isolated and propagated as described^33^. Maintenance of p19^ARF−/−^ hepatocytes and *in vitro* experiments are described in the Supplementary materials and methods (see Supplementary information).

### Statistics

All values are represented as means ± standard error of the mean if not indicated otherwise. Statistical significance was evaluated using a confidence interval of 95% with either one-way ANOVA followed by Tukey’s, Dunns’ or Bonferroni’s post-hoc test for multiple comparison or two-tailed student’s t-test for the comparison of two groups. Differences between experimental groups were considered significant at *p < 0.05, **p < 0.01 and ***p < 0.001. All calculations were performed using GraphPad Prism software (La Jolla, CA).

## Additional Information

**How to cite this article**: Themanns, M. *et al*. Hepatic Deletion of Janus Kinase 2 Counteracts Oxidative Stress in Mice. *Sci. Rep.* **6**, 34719; doi: 10.1038/srep34719 (2016).

## Supplementary Material

Supplementary Information
