# Supplementary material for: Hepatic Deletion of Janus Kinase 2 Counteracts Oxidative Stress in Mice
Source: Sci Rep. 2016 Oct 7;6:34719. doi: 10.1038/srep34719 (PMC5054456; doi:10.1038/srep34719)
Supplement: Supplementary Information [file srep34719-s1.pdf]

Hepatic Deletion of Janus Kinase 2 Counteracts Oxidative Stress in Mice

Madeleine Themanns, Kristina M. Mueller, Sonja M. Kessler, Nicole Golob-Schwarzl, Thomas Mohr, Doris Kaltenecker, Jerome Bourgeais, Jamile Paier-Pourani, Katrin Friedbichler, Doris Schneller, Michaela Schlederer, Eva Zebedin-Brandl, Luigi M. Terracciano, Xiaonan Han, Lukas Kenner, Kay-Uwe Wagner, Wolfgang Mikulits, Andrey V. Kozlov, Markus H. Heim, Fabrice Gouilleux, Johannes Haybaeck, and Richard Moriggl

Supplementary Methods:

Fatty acid measurement by gas chromatography–mass spectrometry (GC-MS). Analysis of hepatic fatty acids was performed using the fatty acid methyl ester (FAME) method. Cryopreserved liver samples were lyophilised, dispersed in 500 µl methanol/toluene/sulfuric acid (50:50:2) [v/v/v], incubated at 55°C overnight, and neutralised by 400 µl of a 0.5 M NH_4CO_3, 2 M KCl solution. After centrifugation the supernatant was derivatised with 25 µl N-methyl-N-(trimethylsilyl)trifluoroacetamide at 37°C for 1 h, and subsequently analysed on a Thermo Fisher Focus gas chromatograph coupled to an DSQII mass selective detector (Fisher Scientific, Schwerte, Germany) equipped with a non-polar J&WDB-5HT capillary column. Methyl-nonadecanoate (74208; Sigma Aldrich) was used as an internal standard.

Reactive Oxygen Species Measurement in liver tissue. Freshly cut livers were placed into ice-cold custodiol solution (Koehler Chemie, Bensheim, Germany) until slice preparation. Cylindrical tissue cores of 6 mm diameter were punched from the left lateral liver lobe and embedded in 3% agarose gel. Slices of 180 µm thickness were cut in ice-cold custodiol solution using a microtome (Compresstome VF-300, Precisionary Instruments Inc., Greenville, NC, USA). Liver slices were placed in Lab-Tek two-chambered cover glasses (Nalge Nunc) and

incubated in Coon's F-12 medium (Sigma) for 10 minutes at 37°C. For laser scanning microscopy liver slices were stained simultaneously with fluorescent dyes sensitive to mitochondrial membrane potential (MitoTracker Deep Red 633, ex/em 630/662 nm), mitochondrial ROS (CM-H2XROS, ex /em 550/599 nm), and cytoplasmic ROS (DCF-DA ex /em: 492/517–527 nm). Staining was performed according to the manufacturer's protocol (Invitrogen). Thereafter, liver slices were transferred on cover glasses (Carlroth) and fixed to the optical table. Imaging was performed with an inverted confocal microscope (LSM 510, Zeiss, Germany) and x630 oil immersion objective. Image analysis was carried out with the histogram toolbar (LSM 510, Zeiss, Germany). Analysis was performed on hepatocytes excluding hepatic infiltrates. Absolute fluorescence intensity was defined as Mean x Area + Area x Threshold.

Transmission electron microscopy. For electron microscopy mouse livers were cut into 2 mm pieces and fixed overnight in 1.6% glutaraldehyde. Pictures were made with a magnification at 8,000x using a transmission electron microscope.

Histology and immunohistochemistry and quantification. Sections prepared from paraffin-embedded formalin-fixed organ specimens were stained with hematoxylin-eosin (H&E). Immunohistochemistry was performed for pH2AX (#9718; Cell Signalling, Danvers, MA) and Ki67 (KI67P-CE, Leica Biosystems, Nussloch, Germany) using standard protocols. Light microscopic images were captured with a PixeLINK camera and the corresponding acquisition software on a Zeiss Imager Z.1. pH2AX and Ki67 positive hepatocytes were quantified using the HistoQuest software (TissueGnostics GmbH, Vienna, Austria; www.tissuegnostics.com). All quantifications were performed with 10 fields/liver from 5-10 mice/genotype.

Immunoblotting. Sample preparation and Western blotting was performed using standard techniques. Membranes were incubated with antibodies against the following proteins: pY-STAT5A/B (#71-6900, Invitrogen, Camarillo, CA); STAT1 (#610115; BD, San Diego, CA); CD36 (ab133625; Abcam, Cambridge, UK); pY-STAT3 (#9131), pY-STAT1 (#7649), JAK2 (#3230), SCD1 (#2794), PPARγ (#2435), FAS (#3180), pY-SRC (#2113), SRC (#2109), RARα (#2554), RXRα (#3085), pT/Y-pERK1/2 (#9106), ERK1/2 (#4695), pS-AKT (#3787; all from Cell Signalling, Danvers, MA); STAT5A/B (N20, #836), STAT3 (H190, #7179), AKT1/2/3 (H136, #8312), GSTA1/2 (sc-398714), PPARα (N19, #1985) and HSC70 (sc-7298, loading control; all from Santa Cruz, CA). Quantification of Western blots was performed using ImageJ.

Quantitative Reverse Transcription Polymerase Chain Reaction (qRT-PCR).

Fig. 4d, 5a,d, Supplementary Fig. S4c

1 µg of total RNA from control and mutant animals (n≥5/genotype) was reverse-transcribed into complementary DNA using the Revert Aid cDNA synthesis kit (Thermo Fisher Scientific). qRT-PCR was performed using the SYBR green method. mRNA levels were normalised for glyceraldehyde 3-phosphate dehydrogenase (*Gapdh*), and relative abundance was calculated using the ΔCt method (gene-specific expression level relative to that of an endogenous housekeeping gene). Each reaction condition was performed in triplicate. Primer sequences are listed in Supplementary Table 1.

Fig. 4b, Supplementary Fig. S4a

RNA samples were reverse-transcribed using SuperScript®VILO cDNA Synthesis kit (Invitrogen) as recommended by the supplier. PCR primers were designed with the ProbeFinder

software (Roche Applied Sciences) and used to amplify the RT-generated cDNAs. PCR reaction mixtures contained equal amounts of cDNAs, primers (0.25 µM), TaqMan mix «LightCycler® 480 Probes Master» (Roche), UPL probe (Universal Probe Library ,Roche) in a final volume of 10 µl. qRT-PCR analysis were performed on the Light Cycler 480 thermocycler II (Roche). Both *Gapdh* and ribosomal Protein L13a (*Rpl13a*) were used as reference genes for normalisation of qRT-PCR experiments. Each reaction condition was performed in triplicate. Relative gene expression was analysed using the ΔCt method. Primer sequences are listed in Supplementary Table 1.

Microarray analysis. Genes for clustering and construction of heatmaps were selected according to the gene ontology and literature. Gene expression was normalised by calculating the Z-score across genes and genes were clustered using pearsons correlation coefficient with average linkage. Clustering and heatmap construction was done using R.

Cell Culture. Hepatocytes of p19$^{ARF-/-}$ mice were isolated and propagated as described[1]. p19$^{ARF-/-}$ hepatocytes (we used passage 10-30) are proficient to lower growth-suppressive functions of p53 and due to p19 deficiency primary cells can bypass cellular senescence. p19$^{ARF-/-}$ hepatocytes were grown on collagen-I coated culture dishes in DMEM supplemented with 10% FCS, 100 U/ml penicillin, 100 mg/ml streptomycin, 2 mM L-glutamine, 35 ng/ml insulin (Novo Nordisk, Mainz, Germany), 20 ng/ml transforming growth factor (TGF)-α (Sigma, St. Louis, MO) and 30 ng/ml insulin-like growth factor (IGF)-II (Sigma).

GH and ruxolitinib treatment

Hepatocytes were treated for 20 minutes with 0.5 µg/ml human growth hormone (hGH) (ImmunoTools, Friesoythe, Germany) or with 3 µM ruxolitinib (CaymanChemical, Michigan,

USA) at indicated time points. Ruxolitinib was freshly dissolved in DMSO at a concentration of 5 mM for individual kinetics. In all controls, equal amounts of DMSO were added to media.

Small interfering (si)RNA-mediated Jak2 *knockdown*

p19$^{ARF-/-}$ hepatocytes (1×10^5 cells) were transfected with 100 nM of murine *Jak2* siRNA and non-targeting siRNA according to manufacturer's protocol (murine si*Jak2*: #L-040118-00-0005; non-targeting siRNA: #D-001810-10-05, Dharmacon). After 48 hours of transfection by lipofectamine (RNAiMAX, Life Technologies), knockdown efficiency was confirmed by qRT-PCR and Western blot analysis. Additionally, transcriptional expression of *Gstα1*, *Gstα2*, *Gstm3* and *Cd36* was determined 48 hours after transfection.

Palmitic acid- and H_2O_2-induced DNA damage

A 50 mM palmitic acid (PA, Sigma #P0500) stock solution was prepared in ethanol by heating for 10 minutes at 60°C in a water bath and occasional vortexing. Simultaneously, a 10% (w/v) FFA-free BSA (Sigma #A8806) solution was prepared in PBS and maintained at 40°C in a water bath. A 5 mM PA solution was obtained by complexing the appropriate amount of PA stock solution to 10% BSA. Briefly, the PA stock solution was added drop wise to 10% BSA solution at 40°C in a shaking water bath (200 rpm), followed by 30 minutes incubation at 40°C (200 rpm). The PA/BSA complex solution was cooled to RT and stored at −20°C until use. Before use, the PA/BSA complex solution was heated for 10 minutes at 60°C, vortexed occasionally and then cooled to RT. The control solution containing equal amounts of ethanol and BSA was prepared similarly. For all experimental procedures, cells were seeded in collagen-coated 4-well chamber slides at $5\text{x}10^4$ cells per well 12 h prior to experiments.

<u>PA-induced oxidative DNA damage:</u> i) 24 hours ruxolitinib (3 µM, 0.06% DMSO) or DMSO (0.06%) pre-treated cells, were treated for another 24 hours with 300 µM PA/0.28% BSA solution containing either ruxolitinib or DMSO. ii) After 48 hours, si*Jak2* (100 nM) or si*Control*

(100 nM) pre-treated cells were washed and treated for another 24 hours with 300 µM PA/0.28% BSA solution.

<u>H_2O_2-induced oxidative DNA damage:</u> i) 46 hours ruxolitinib (3 µM, 0.06% DMSO) or DMSO (0.06%) pre-treated cells, were treated for another 2 hours with 25 µM H_2O_2 (Sigma #H1009) in presence of either ruxolitinib or DMSO. Control cells were treated similarly with ruxolitinib or DMSO. ii) After 48 hours, si*Jak2* (100 nM) or si*Control* (100 nM) pre-treated cells were washed and treated for another 2 hours with 25 µM H_2O_2 (Sigma #H1009).

After the indicated treatments, cells were washed twice with PBS, fixed with 4% (vol/vol) formaldehyde for 15 minutes at room temperature followed by three washes with PBS (pH 8,5 min each). Slides were blocked for 1 hour at room temperature in blocking buffer (PBS pH 8 / 5% donkey serum / 0.3% Triton X-100) before incubated with pH2AX antibodies (1:400, Cell Signalling #9718) overnight at 4°C temperature in antibody dilution buffer (PBS pH 8 / 1% BSA / 0.3% Triton X-100). Slides were washed three times in PBS before incubation in the dark with Alexa Fluor 488-conjugated secondary antibodies (1:400 in antibody dilution buffer, Invitrogen, A21206) for 2 hours at room temperature. After three washes in PBS, slides were mounted in Vectashield media containing DAPI (4′,6-diamidino-2-phenylindole; Vector Laboratories). Cell imaging and data collection (20x magnification) was performed with an EVOS FL Cell Imaging System (ThermoFisher). The exposure length and gain were maintained at a constant level for all samples. Quantification of pH2AX positive cells was performed using ImageJ.

Mice. All mice were kept under standardised conditions at the Decentralised Biomedical Facilities, Medical University of Vienna (Vienna, Austria). Mice were kept on a 12 hours light-dark cycle and fed a standard diet (#V1126-000, ssniff Spezialdiäten GmbH, Soest, Germany).

The nutrient composition as indicated by the manufacturer was: 22.1% raw protein, 4.5% raw fat, 46.8% starch and 10.8 % glucose. For some experiments control mice not expressing AlfpCre recombinase were treated with vehicle (5% DMSO, 0.5% methyl cellulose (Sigma, St. Louis, MO)) or 30 mg/kg JAK inhibitor ruxolitinib (INCB018424, Selleckchem, Houston, TX) twice daily by oral gavage for 2 consecutive days. Furthermore, WT and JAK2$^{\Delta ep}$ mice were injected intraperitoneally with vehicle (PBS) or 2 mg/kg recombinant hGH (Immunotools, Friesoythe, Germany) and sacrificed at indicated time points.

Supplementary Table, Figures and Figure Legends:

Supplementary Table S1. Primer sequences used for qRT-PCR analysis.

Overview of exon-spanning primers which were used for qRT-PCR analysis.

Gene	Murine primers
Gsta1	for: TGGAGAAGAAGCCAGGACTC<br>rev: CTCTTCAAACTCCACCCCTG
Gsta2	for: CACTCCTCTGGAGCTGGATT<br>rev: CCGGGCATTGAAGTAGTGAA
Gstm3	for: CGTATGTTTGAGCCCAAGTG<br>rev: AACACAGGTCTTGGGAGGAA
Nrf2	for: TGCTCCTATGCGTGAATC<br>rev: CGACAGGGAATGGAATATGG
Cd36	for: CCTTACTTGGGATTGGAGTGG<br>rev: CGGCTTTACCAAAGATGTAGCC
Gapdh	for: AGAAGGTGGTGAAGCAGGCATC<br>rev: CGGCATCGAAGGTGGAAGAGTG

Gene	Murine primers	UPL probes
Cat	for: GCGACCAGATGAAGCAGTG<br>rev: GTGGTCAGGACATCAGGTCTC	#68
Gpx1	for: TTTCCCGTGCAATCAGTTC<br>rev: TCGGACGTACTTGAGGGAAT	#2
Gpx2	for: GTTCTCGGCTTCCCTTGC<br>rev: TCAGGATCTCCTCGTTCTGAC	#2
Gpx3	for: GGCTTCCCTTCCAACCAA<br>rev: CCCACCTGGTCGAACATACT	#92
Gpx4 (1)	for: CGAGTTCCTGGGCTTGTG<br>rev: TTATCCAGGCAGACCATGTG	#102
Gpx6	for: CTTCTGTGGCCTGACAGCTA<br>rev: CGTGACGTTGAATGGCTTC	#63
Gpx7	for: CACCCTGCCTTCAAGTACCTA<br>rev: TTTCCGTCTGGGTCCACTA	#12
Gss	for: CTCGGAGCTGGGTATTTTTG<br>rev: GCTTTGGTTCGAAGCAGGT	#9
Hmox1	for: AGGCTAAGACCGCCTTCCT<br>rev: TGTGTTCCTCTGTCAGCATCA	#17
Sod1	for: CAGGACCTCATTTTAATCCTCAC<br>rev: TGCCCAGGTCTCCAACAT	#49
Sod3	for: CTCTTGGGAGAGCCTGACA<br>rev: GCCAGTAGCAAGCCGTAGAA	#102
Prdx1	for: GTGAGACCTGTGGCTCGAC<br>rev: TGTCCATCTGGCATAACAGC	#15
Gapdh	for: TGTCCGTCGTGGATCTGAC<br>rev: CCTGCTTCACCACCTTCTTG	#80
Rpl13a	for: CATGAGGTCGGGTGGAAGTA<br>rev: GCCTGTTTCCGTAACCTCAA	#25

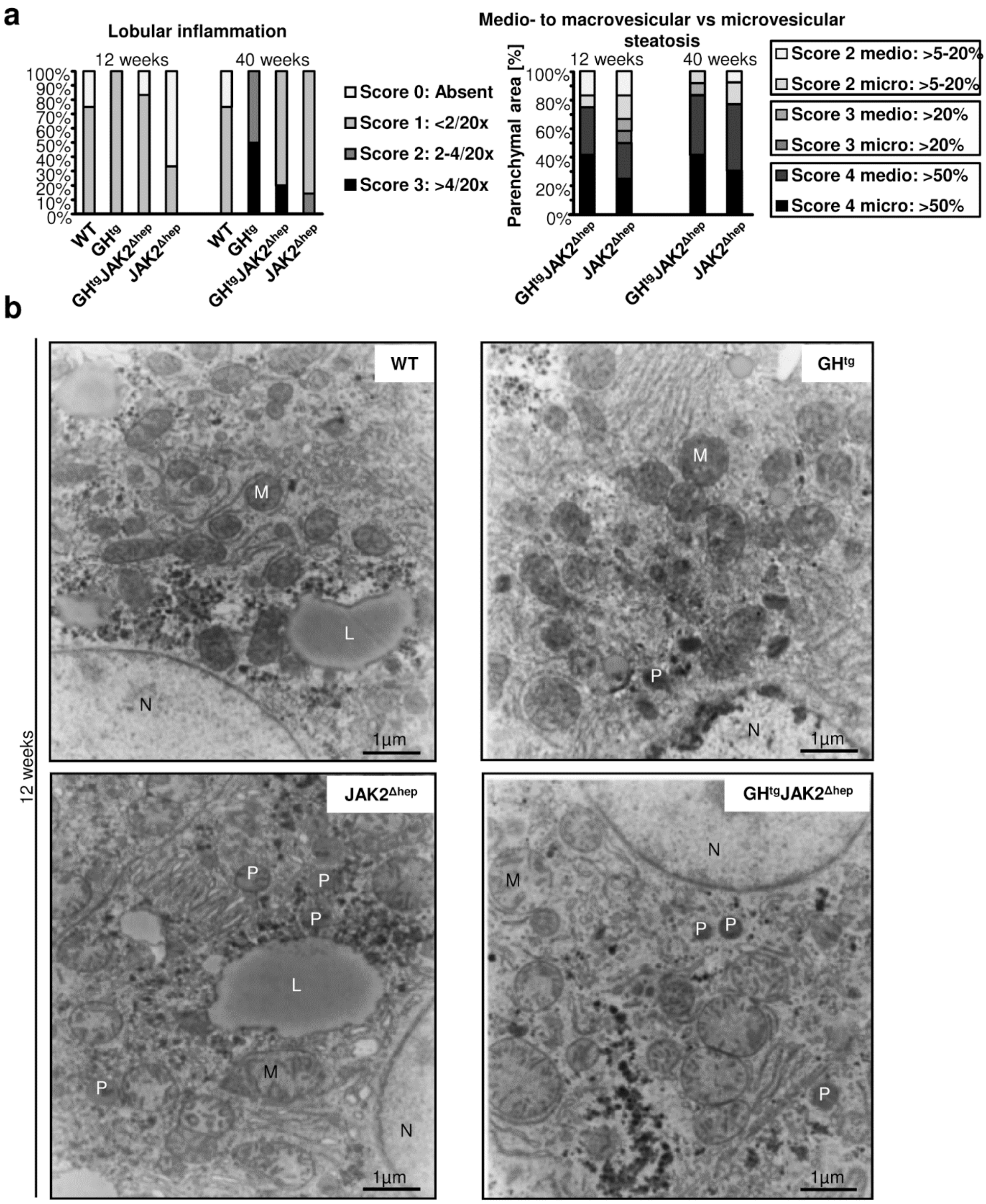

Supplementary Figure S1. Characterization of liver histopathology.

(a) Lobular inflammation was assessed in H&E-stained liver sections. Histopathological analysis of medio- to macrovesicular steatosis in relation to microvesicular steatosis (n≥4/genotype). (b) Transmission electron microscopy (8000x magnification) in livers of 12-

week-old mice (n=3/genotype). GH^{tg} mice revealed large nuclei with irregular shape. Upon deletion of JAK2, hepatocytes had normal shaped nuclei but displayed large and swollen mitochondria (N: nucleus, L: lipids, M: mitochondria, P: peroxisome).

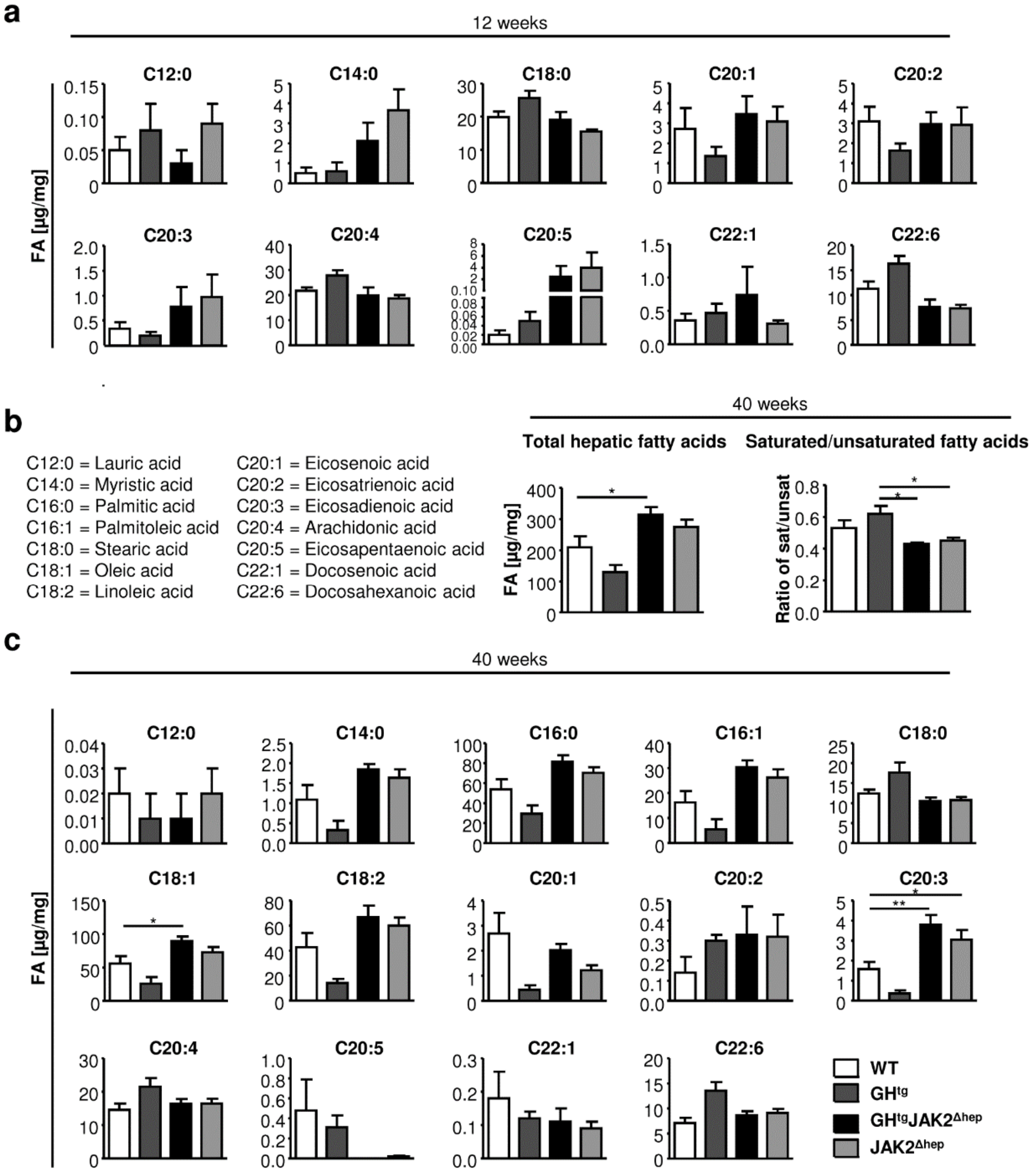

Supplementary Figure S2. Assessment of hepatic fatty acids.

(a) At 12 weeks of age, hepatic fatty acids were measured by gas chromatography–mass spectrometry (GC-MS) (n≥8/genotype). (b) FA amounts at 40 weeks of age. (C) Detailed profiling of FAs in steatotic livers using GC-MS (n≥8/genotype). * $p < 0.05$.

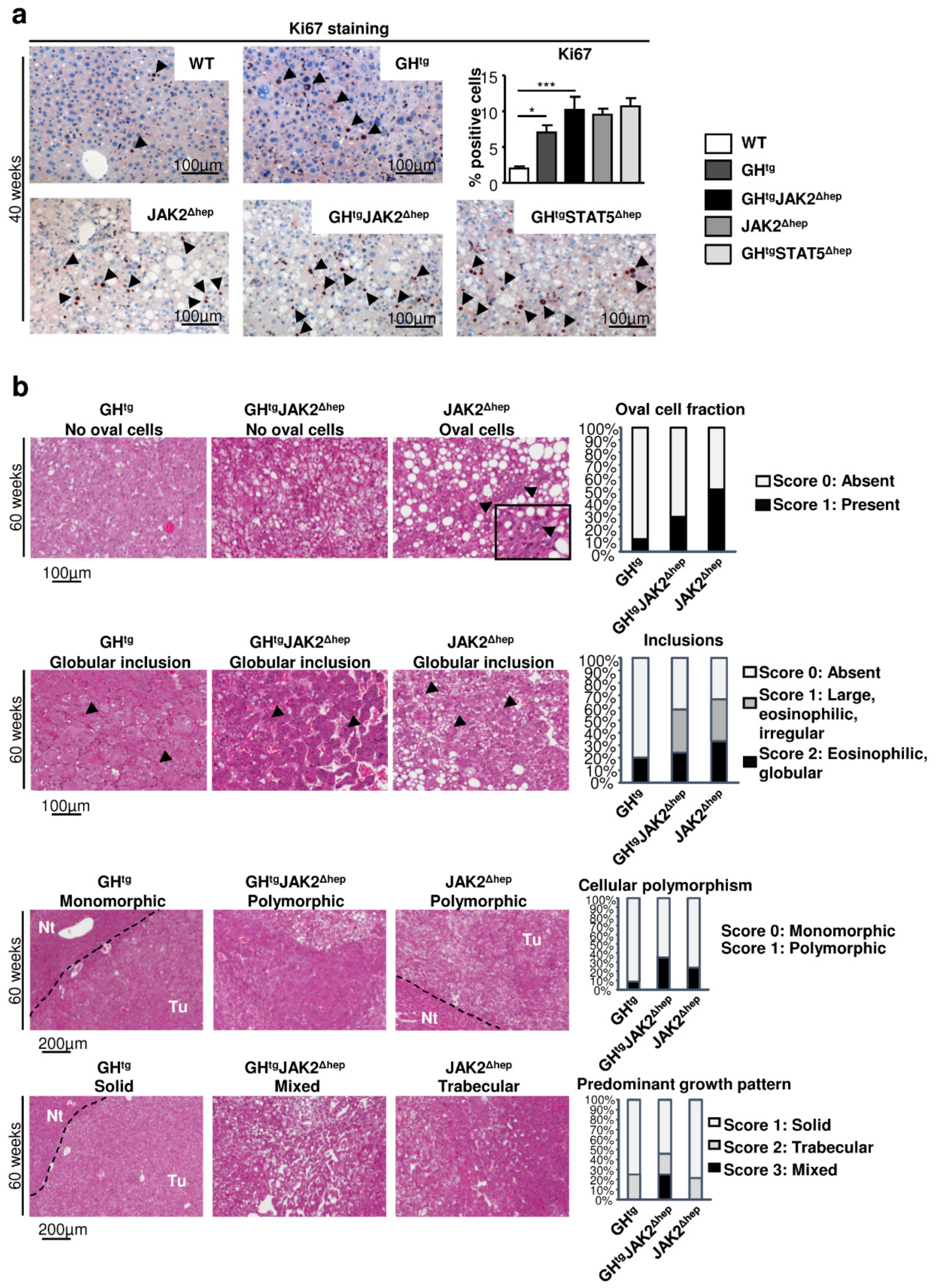

Supplementary Figure S3. Liver tumour characterization.

(a) Liver sections of 40-week-old animals stained with antibodies against Ki67 showed increased proliferation in GH^{tg}, $GH^{tg}JAK2^{\Delta hep}$, $JAK2^{\Delta hep}$, and $GH^{tg}STAT5^{\Delta hep}$ mice

(n≥5/genotype). (b) At 60 weeks of age, percentage distribution profiling revealed that tumours from JAK2 deficient livers displayed increased marker for malignancy (oval fraction, predominant inclusions) (n≥4/genotype). At 60 weeks histopathological analysis revealed that tumours from GH^{tg} mice were less malignant compared to JAK2-deficient tumours. Tumour grading showed that $GH^{tg}JAK2^{\Delta hep}$ and $JAK2^{\Delta hep}$ mice develop more frequently tumours with cellular polymorphism and tumours with solid and trabecular growth pattern than GH^{tg} animals (n≥4/genotype).* $p < 0.05$ and *** $p < 0.001$.

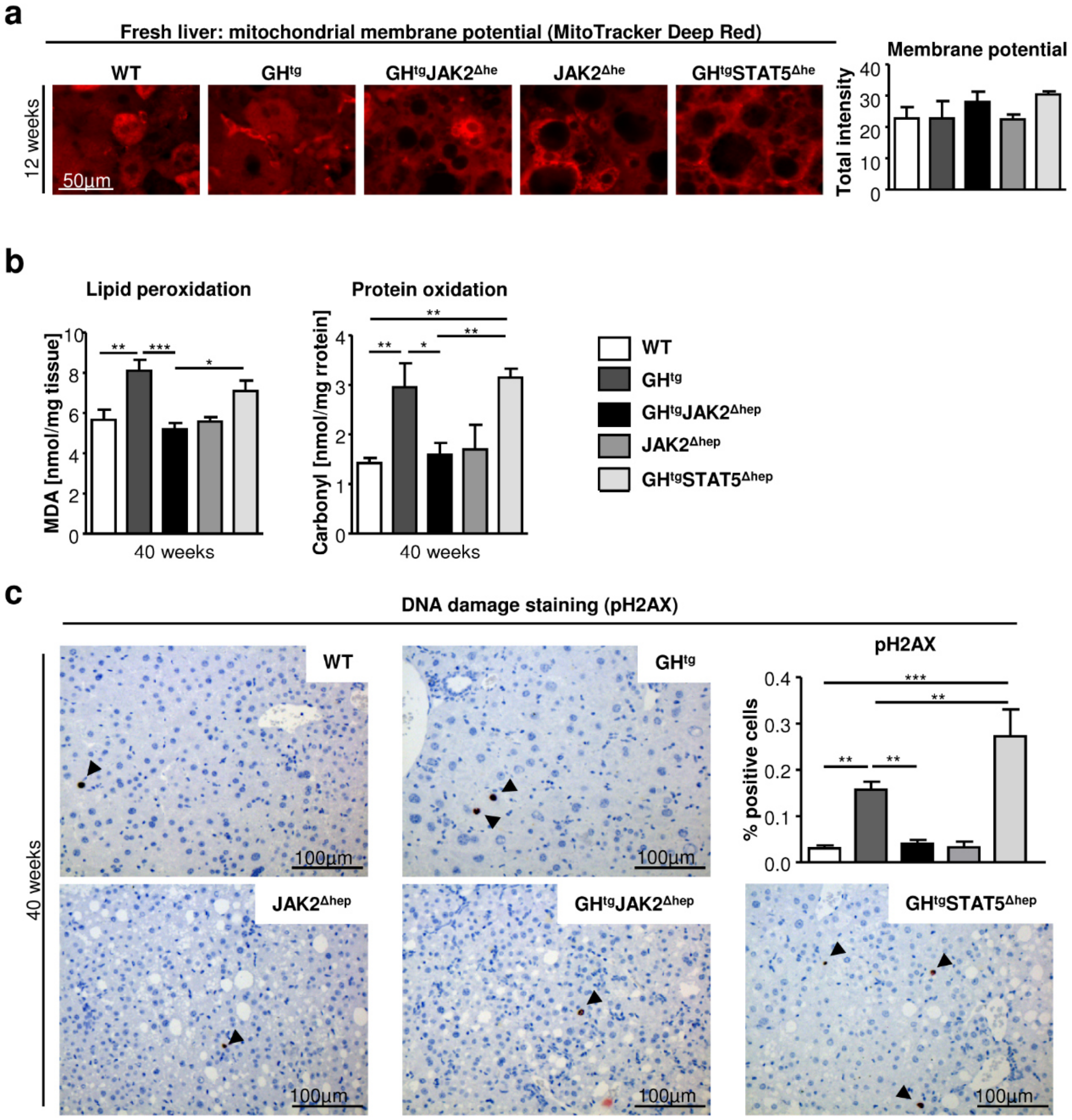

Supplementary Figure S4. Hepatic JAK2 deficiency protects against ROS-induced lipid peroxidation, protein oxidation and DNA damage.

(a) At 12 weeks, freshly cut livers were stained with fluorescent dyes sensitive to mitochondrial membrane potential. Imaging was performed with an inverted confocal microscope (n≥5/genotype). (b) Lipid peroxidation was assessed by measuring its by-product malondialdehyde (MDA) at 40 weeks of age. Protein oxidation was performed using a colorimetric assay (n≥5/genotype). (c) Representative liver sections of 40-week-old mice stained with antibodies against phosphorylated H2AX to detect DNA damage. Quantification of positive hepatocytes by image analysis (n≥ 5/genotype). * $p < 0.05$, ** $p < 0.01$ and *** $p < 0.001$.

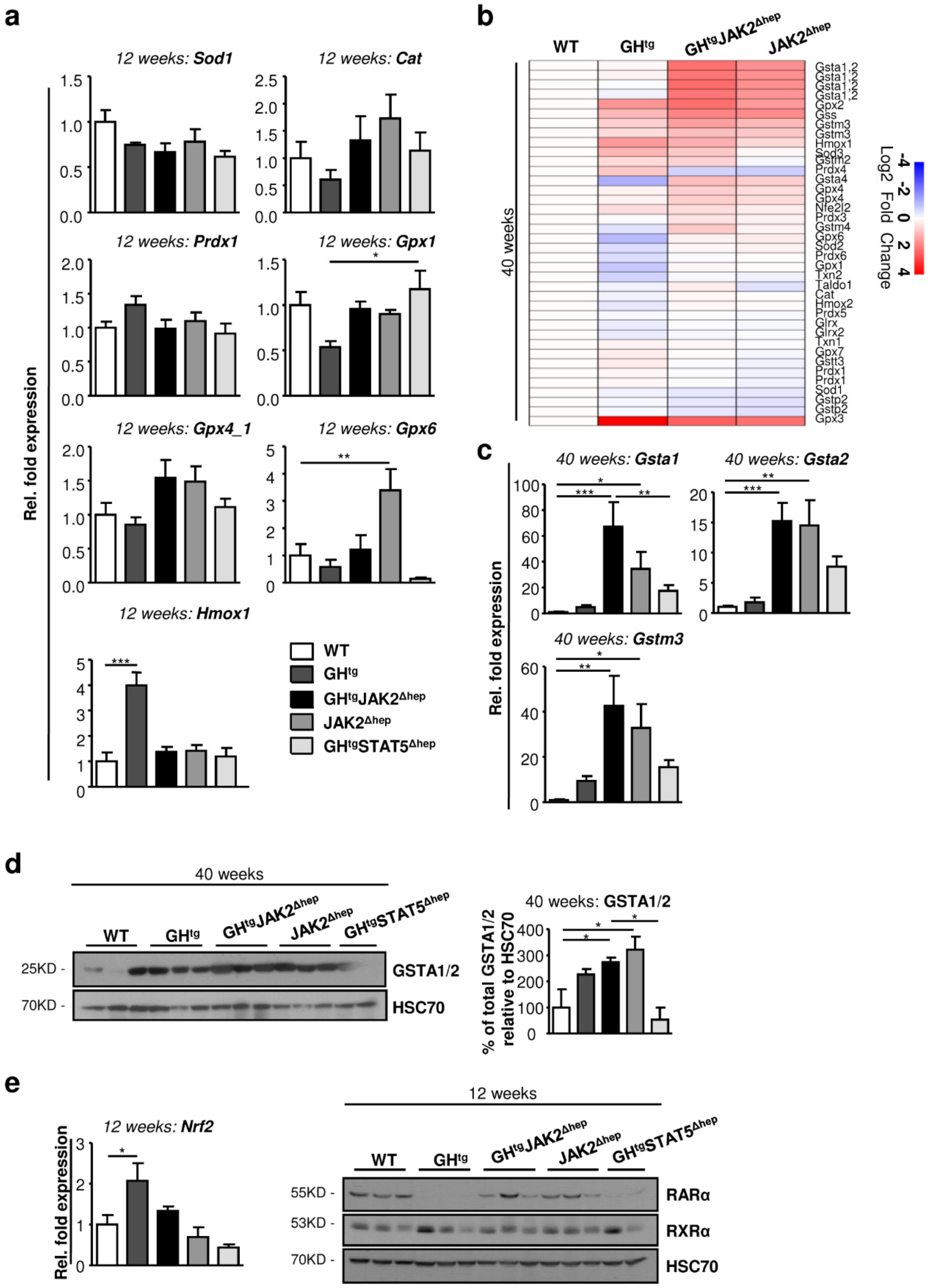

Supplementary Figure S5. Analysis of the antioxidant defence system.

(a) By means of qRT-PCR, expression *Sod1*, *Cat*, *Prdx1*, *Gpx1*, *Gpx4_1*, *Gpx6* and *Hmox1* were assessed at 12 weeks of age (n=6/genotype). Ct values were normalised to *Gapdh* and

Rpl13a. (b) At 40 weeks of age transcriptome analysis of genes coding for enzymes involved in antioxidant defence. (c) mRNA expression levels of *Gsta1*, *Gsta2* and *Gstm3* at 40 weeks of age (n=6/genotype). Ct values were normalised to *Gapdh*. (d) Representative Western blot analysis of whole liver homogenates and Western blot quantification of GSTA1/2 from 40-week-old animals. As a loading control HSC70 is shown (n≥2/genotype). (e) By means of qRT-PCR, mRNA levels of *Nrf2* were measured in livers at 12 weeks of age (n=6/genotype). Representative Western blot analysis of whole liver homogenates from 12-week-old animals. As a loading control HSC70 is shown (n≥2/genotype). * $p < 0.05$, ** $p < 0.01$ and *** $p < 0.001$.

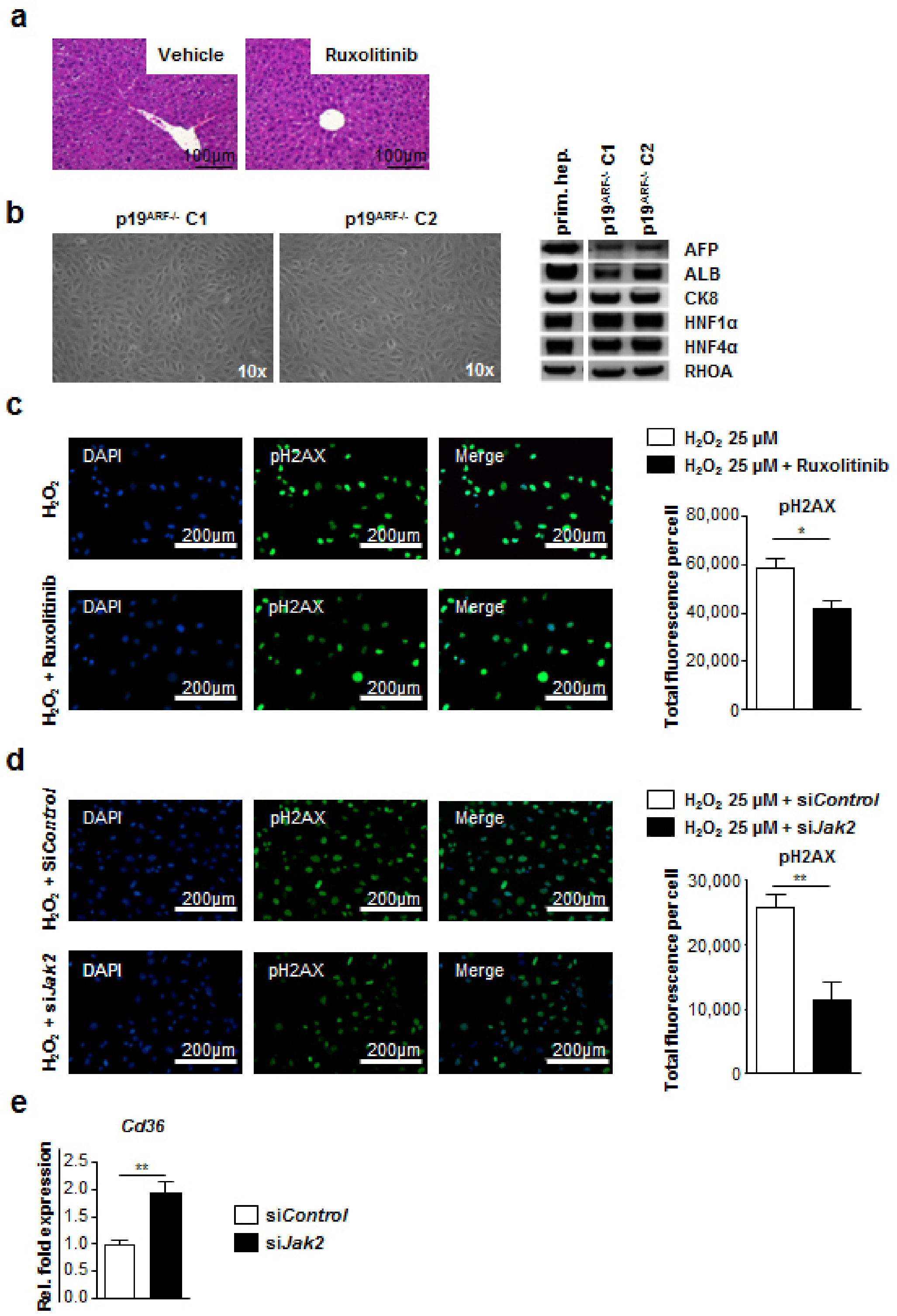

Supplementary Figure S6. Expression of liver-specific markers and H_2O_2-induced DNA-damage in immortalised hepatocytes.

(a) H&E-stained liver sections showed normal liver architecture in ruxolitinib-treated mice. (b) Optical microscopy images of two different p19$^{ARF-/-}$ hepatocyte clones. Monitoring of hepatocellular marker expression. Representative Western blot analysis of two different hepatic p19$^{ARF-/-}$ clones. Primary hepatocytes were shown as a positive control. As loading control RHOA is shown (AFP: α-fetoprotein, ALB: albumin, CK8: Cytokeratin 8, HNF: hepatocyte nuclear factor; analysis was performed on one membrane). (c) Immunofluorescence staining of DAPI (blue) and phosphorylated H2AX (green). p19$^{ARF-/-}$ hepatocytes were pre-treated 24 hours with ruxolitinib or DMSO. For another 2 hours, cells were exposed with 25 µM H_2O_2 containing either ruxolitinib or DMSO. Fluorescence intensity was quantified by image analysis (n=3 independent experiments). (d) Immunofluorescence staining of DAPI (blue) and phosphorylated H2AX (green). p19$^{ARF-/-}$ hepatocytes were pre-treated 48 hours with si*Jak2* or si*Control*. For another 2 hours, cells were exposed with 25 µM H_2O_2. Fluorescence intensity was quantified by image analysis (n=2 independent experiments). (e) p19$^{ARF-/-}$ hepatocytes were transfected with si*Jak2* or si*Control* for 48 hours. mRNA expression levels of *Cd36* upon siRNA-mediated knockdown were measured. Ct values were normalised to *Gapdh*. * $p < 0.05$ and ** $p < 0.01$.

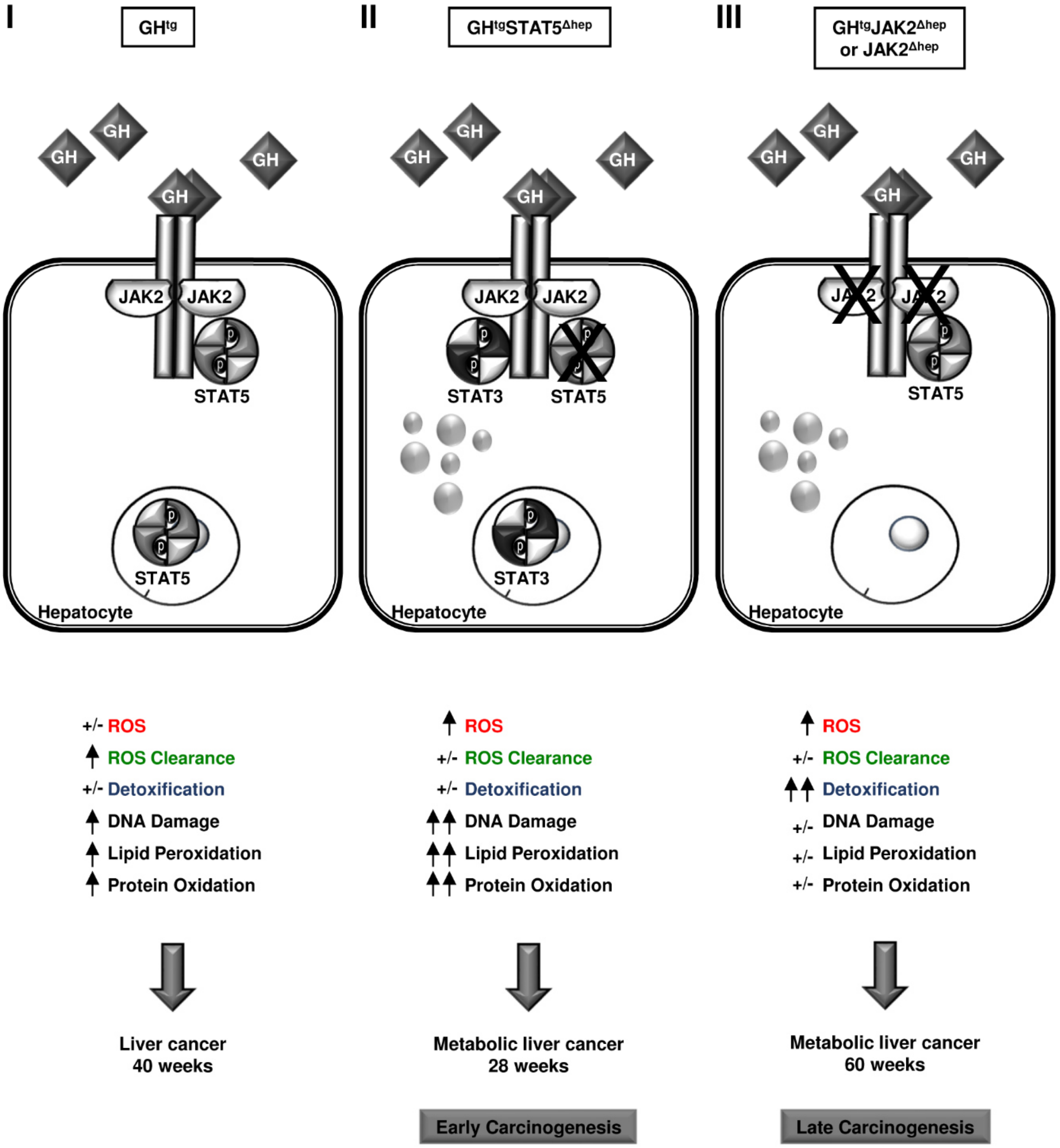

Supplementary Figure S7. Schematic demonstration illustrating the development of phenotypes following hepatic STAT5 and JAK2 deletion in GH^{tg} mice.

(I) Exposure of GH leads to the activation of the JAK2-STAT5 signalling pathway. GH^{tg} mice develop their first liver tumours at 40 weeks of age which can be explained by increased regeneration and proliferation. Despite increased extracellular clearance of ROS via GH-dependent expression of the antioxidants SOD3, GPX2, 3 and 7, constant GH signalling leads

to accumulation of DNA damage, lipid peroxidation and protein oxidation within hepatocytes contributing to tissue damage and tumour formation. (II) Additional loss of STAT5 induces FLD and strongly accelerates tumour development and progression at 28 weeks of age by enhancing the activity of STAT3. Secondary, deletion of STAT5 leads to increased production of ROS which causes pronounced DNA damage, lipid peroxidation and protein oxidation due to inadequate protection mechanisms against ROS. Consequently, loss of STAT5 promotes the development of steatotic tumours. (III) On the contrary, despite steatosis formation, loss of hepatic JAK2 results in diminished tumour formation, which is associated with diminished oxidative damage as compared to $GH^{tg}STAT5^{\Delta hep}$ mice, despite equally severe steatosis and ROS formation. In addition, the reduced oxidative damage in JAK2-deficient livers is linked to increased expression and activity of detoxifying enzymes. We conclude that reduced ROS protection is associated with JAK2 expression and activity, which causes increased oxidative damage.

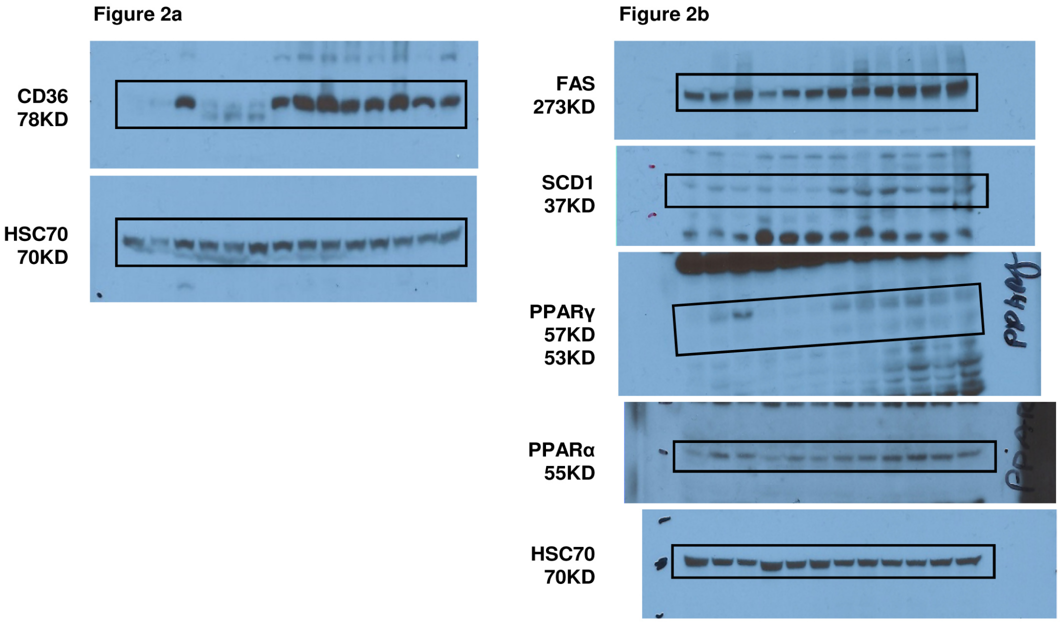

Supplementary Figure S8. Uncropped scans of Western blots shown in Figure 2.

Original images of Western blots. Boxed areas indicate the cropped regions.

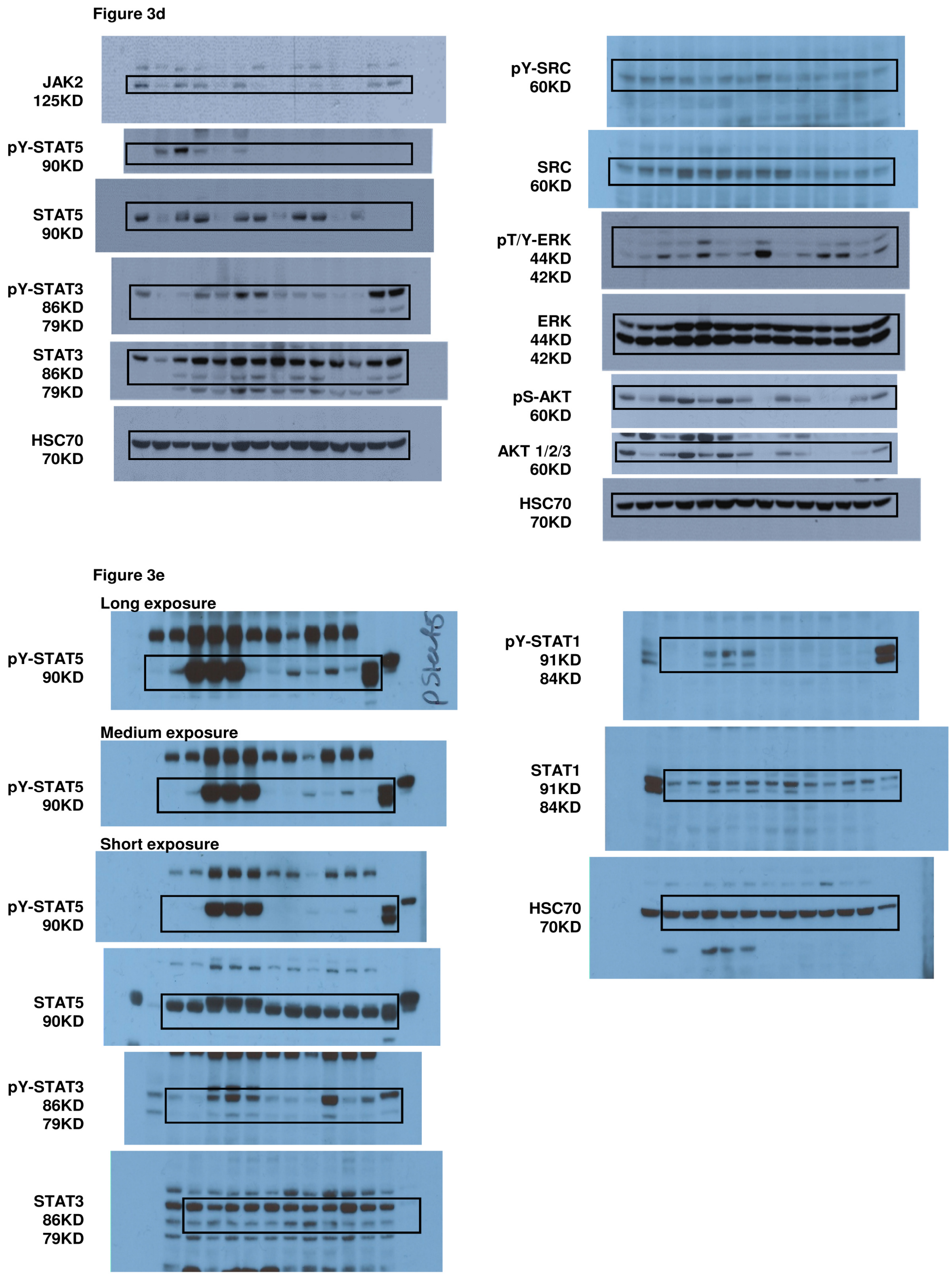

Supplementary Figure S9. Uncropped scans of Western blots shown in Figure 3.

Original images of Western blots. Boxed areas indicate the cropped regions.

Figure 5e

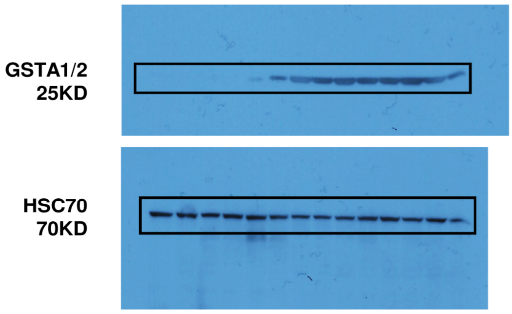

Supplementary Figure S10. Uncropped scans of Western blots shown in Figure 5.

Original images of Western blots. Boxed areas indicate the cropped regions.

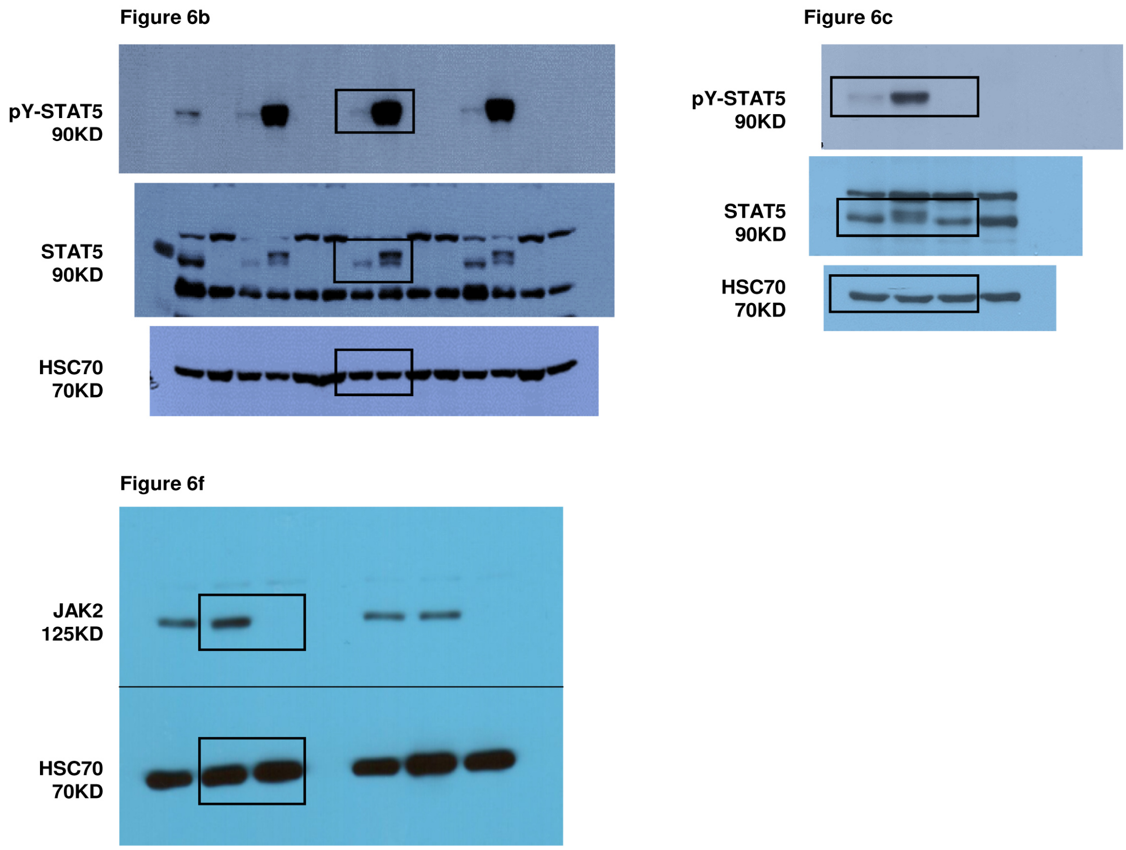

Supplementary Figure 11. Uncropped scans of Western blots shown in Figure 6.

Original images of Western blots. In Fig. 6f membrane was cut prior to antibody staining for the detection of proteins running at different sizes on the same membrane. Boxed areas indicate the cropped regions.

Supplementary References:

1 Mikula, M. *et al.* Immortalized p19ARF null hepatocytes restore liver injury and generate hepatic progenitors after transplantation. *Hepatology* **39**, 628-634, doi:10.1002/hep.20084 (2004).
